# Multilevel analysis of salt stress responses in sorghum during seed germination

**DOI:** 10.3389/fpls.2026.1802398

**Published:** 2026-03-19

**Authors:** Hong-jin Wang, Yi Yu, Yun Zhao, Hui Wang, Xiangwei Hu, Uzair Ullah, Baoyi Yang, Jianan Huang, Kunming Chen, Aamir Hamid Khan, Waseem Abbas, Guojun Feng, Zaituniguli Kuerban

**Affiliations:** 1 Crop Research Institute of Xinjiang Uygur Autonomous Region Academy of Agricultural Sciences, Urumqi, Xinjiang, China; 2 National Key Laboratory of Crop Improvement for Stress Tolerance and Production, College of Life Sciences, Northwest A&F University, Shaanxi, China; 3 College of Pratacultural Science, Xinjiang Agricultural University, Urumqi, Xinjiang, China; 4 Department of Biogeography, Paleoecology and Nature Conservation, Faculty of Biology and Environmental Protection, University of Łódź, Łódź, Poland; 5 Guangdong Provincial Key Laboratory for Plant Epigenetics, Longhua Bioindustry and Innovation Research Institute, College of Life Sciences and Oceanography, Shenzhen University, Shenzhen, China

**Keywords:** antioxidant defense, ion homeostasis, salt tolerance, seedling vigor, sorghum

## Abstract

A significant number of crop species and their associated agricultural landscapes are impacted by soil salinity, which severely hampers germination and seedling establishment. Developing salt-tolerant germplasm and improving screening methods are critical priorities for plant breeding programs. This study used sorghum as a model to evaluate germination and early growth responses in 100 accessions under control and 150 mM NaCl conditions. Multiple indices were used to develop a numeric salt tolerance score (STS), minimizing baseline growth effects to better reflect true stress responses. Multivariate approaches, including PCA, cluster analysis, and machine learning, were employed to assess salt tolerance. Salt stress significantly reduced germination traits such as germination index, rate, and vigor. The STS proved effective in ranking accessions for salt tolerance. Salt-tolerant accession showed upregulation of ion transport and homeostasis genes (*SbNHXLP*, *SbHKT1;4*, *SbHKT1;5*, and *SbCLCc*), and downregulation of certain transcription factors. Physiological analyses revealed lower sodium accumulation, higher K+/Na+ ratios, and enhanced antioxidant and osmotic regulation in tolerant accessions. These findings suggest that coordinated ion homeostasis and stress responses are likely determinants of salt tolerance during germination. This integrated screening system offers a valuable tool for early-stage breeding of salt-tolerant sorghum.

## 1 Introduction

Soil salinization is one of the most severe abiotic stresses affecting global agricultural production, particularly in arid, semi-arid, and irrigated regions (Nikolić et al., 2023; Tarolli et al., 2024). With the intensification of climate change, declining irrigation water quality, and irrational land use, the area of salinized land continues to expand, severely restricting safe crop production and sustainable development (Ullah et al., 2021; Nascimento Araújo Júnior et al., 2023; Tarolli et al., 2024). Salt stress primarily affects plant growth through two mechanisms: osmotic stress and ion toxicity (Zhu, 2002; Munns and Tester, 2008). On the one hand, high salinity reduces soil water potential, thereby inhibiting water uptake by the seed and cell expansion (Panuccio et al., 2014; Arif et al., 2020). On the other hand, excessive accumulation of Na^+^ and Cl^-^ in plants disrupts ion homeostasis, causing metabolic disorders and oxidative damage, thus significantly inhibiting crop germination, growth, and yield formation (Isayenkov and Maathuis, 2019; Hussain et al., 2021). During the entire crop growth cycle, the germination and emergence stages are considered among the most sensitive to salt stress (Ibrahim, 2016). Salt stress can significantly delay germination time, reduce germination rate, and weaken seedling vigor, directly affecting the quality of field colony establishment and subsequent growth potential (Ibrahim, 2016; Manono, 2025). Numerous studies have shown that different crop genotypes exhibit significant differences in their responses to salt stress during germination (Mwando et al., 2020; Farooq et al., 2021). These differences not only reflect the inherent stress resistance potential of the materials but also provide an important screening window for salt-tolerant breeding. Therefore, establishing an efficient, stable, and breeding-guiding salt tolerance evaluation system at the germination stage has significant theoretical and practical value for crop improvement in saline-alkali areas.

Sorghum (*Sorghum bicolor* (L.) Moench), as an important food, feed, and energy crop, possesses characteristics such as drought resistance, tolerance to poor soil, and high biomass, making it a promising candidate for use in marginal lands and saline-alkali lands (Mullet et al., 2014; Mwamahonje et al., 2024). It has been reported that sorghum exhibits significant genotypic differences in its response to salt stress at different growth stages, especially during germination and seedling stages, where different genotypes show marked differentiation in germination ability, early growth potential, and ion homeostasis regulation (Dehnavi et al., 2020; Mansour et al., 2021). However, current research on salt tolerance during sorghum germination mainly focuses on comparative analyses of single or a few traits, such as germination rate, germination potential, or seedling length (Mulaudzi et al., 2020). This evaluation method struggles to comprehensively reflect the overall performance of materials under salt stress and is easily affected by baseline growth differences, thus impacting the stability and reproducibility of screening results.

In recent years, with the development of multi-trait phenotypic analysis and data-driven methods, multi-trait comprehensive evaluation strategies have been gradually introduced into crop stress resistance research (Olivoto et al., 2022; Sheikh et al., 2024). Compared to single-trait threshold screening, multi-trait integration methods can characterize the overall response characteristics of materials under stress conditions, reducing the uncertainty caused by fluctuations in single indicators and improving the robustness of screening results (Olivoto et al., 2022; Pour-Aboughadareh et al., 2025). Especially in breeding practice, evaluation approaches centered on “relative retention capacity” (such as relative trait retention rate, response intensity index, etc.) can effectively mitigate the influence of growth differences under control conditions, more realistically reflecting the material’s response to salt stress (Negrão et al., 2017; Chiradza et al., 2025). However, in studies on salt tolerance during sorghum germination, the systematic construction of a multi-trait comprehensive evaluation framework guided by breeding screening, combined with statistical analysis and model validation, remains relatively limited.

Furthermore, relying solely on phenotypic screening often fails to reveal the intrinsic biological basis of salt tolerance formation. Existing research indicates that salt tolerance is closely related to ion homeostasis regulation, antioxidant defense, and transcriptional regulatory networks (Deinlein et al., 2014; Isayenkov and Maathuis, 2019). The maintenance of Na^+^/K^+^ balance, Na^+^ segregation and transport, and activation of antioxidant enzyme systems are all important mechanisms for plant adaptation to salt stress (Zhu, 2002; Munns and Tester, 2008; Deinlein et al., 2014). However, whether the response characteristics of different salt-tolerant materials at these molecular and physiological levels during sorghum germination are consistent with phenotypic screening results still lacks systematic joint verification.

Previous studies have identified several key biomarkers associated with salt tolerance in sorghum during germination and early growth. These include the physiological indicators such as germination rate, seedling vigor, and biomass retention (Milošević and Vujaković, 2010; Hmissi et al., 2023; Li et al., 2023); biochemical markers such as proline accumulation, antioxidant enzyme activities (SOD, and POD), and reactive oxygen species (ROS) levels (Ahmad et al., 2010; Du et al., 2014; Liu et al., 2014); and molecular markers, including genes involved in ion transport and homeostasis (*SbNHXLP*, *SbHKT1;4*, *SbHKT1;5*, *SbCLCc*) (Wang et al., 2014; Guo et al., 2023; Kang et al., 2024) and transcriptional regulators (*SbTEF1*, *SbMYBHv33*, *SbWRKY50*, *SbbHLH85*) (Zheng et al., 2023; Liu et al., 2024; Chen et al., 2026). These markers collectively reflect the ability of a genotype to maintain water balance, prevent oxidative damage, and regulate ion distribution under salt stress, forming reliable indicators for evaluating salt tolerance in sorghum. Despite extensive research on salt tolerance in sorghum, most previous studies have focused on single traits, such as germination rate or seedling length, with a limited number of genotypes and under specific stress conditions. These approaches are insufficient to capture the complex, quantitative nature of salt tolerance, are sensitive to baseline growth differences, and rarely integrate molecular or physiological mechanisms. Consequently, there is a need for a comprehensive, multi-level evaluation framework that can reliably characterize salt tolerance during germination and support breeding efforts.

In this study, we addressed existing gaps in sorghum salt tolerance research by evaluating 100 sorghum accessions under control and salt (150 mM NaCl) stress conditions, systematically measuring multiple germination and early growth traits. The concentration of 150 mM NaCl was selected based on previous studies (Amzallag, 2005; Wang et al., 2025; Wang et al., 2025), indicating that this level represents moderate to severe salt stress for sorghum during germination, capable of clearly differentiating tolerant and sensitive genotypes without completely inhibiting germination. This concentration has been widely used in sorghum salt tolerance screening studies, providing comparability with existing literature. We constructed a comprehensive multi-trait retention-based Salt Tolerance Score (STS) that captures the ability to maintain relative traits under stress. The STS was validated through principal component analysis, cluster analysis, and machine learning classification, enabling stable differentiation between salt-tolerant and sensitive materials. Based on this framework, extremely salt-tolerant and sensitive accessions were further identified, and key salt stress-related genes and ion homeostasis characteristics were verified at the transcriptional and physiological levels. This integrated approach provides novel insights into the mechanisms underlying salt tolerance and represents a significant advance over prior single-trait or limited-scope studies. Specifically, the study aims to: 1) establish a robust, multi-trait evaluation system for sorghum germination with practical breeding value; 2) systematically characterize phenotypic responses of sorghum accessions with varying salt tolerance; and 3) elucidate key molecular and physiological determinants of salt tolerance during germination. The findings offer a theoretical foundation and methodological reference for screening salt-tolerant sorghum germplasm and guiding early-stage breeding programs.

## 2 Materials and methods

### 2.1 Source of plant materials and experimental design

This study selected 100 sorghums (*Sorghum bicolor* L. Moench) varieties/materials as research subjects. These materials were derived from previous breeding populations and germplasm resources provided by the Crop Research Institute of Xinjiang Uygur Autonomous Region Academy of Agricultural Sciences (XAAS), Urumqi, Xinjiang, China. The selected materials represent diverse genetic backgrounds and were used to systematically evaluate and analyze salt tolerance during germination for breeding applications. All materials were stored at room temperature and dried before the experiment. Seeds with plump grains, uniform size, and free from disease spots and mechanical damage were selected for testing. To ensure the reproducibility and statistical reliability of the experimental results, three biological replicates were set up for all materials under control and salt stress conditions. The experiment employed a completely randomized design with two treatment conditions (Tarolli et al., 2024): Control treatment (CK): distilled water without NaCl (Nikolić et al., 2023); Salt stress treatment (NaCl150): distilled water with NaCl added to a final concentration of 150 mmol·L^-^¹.

### 2.2 Hydroponic germination and salt stress treatment conditions

The salt tolerance experiment during germination was conducted hydroponically to reduce the interference of soil physicochemical differences on phenotypic determination and to ensure precise control of salt concentration (Fellahi et al., 2024). The experiment was conducted in a climate chamber. We selected a constant temperature of 25 ± 1 °C with a 12 h light/12 h dark photoperiod and ~60–70 % relative humidity for germination because this regime reflects the optimum conditions for sorghum seed germination under controlled environments, producing high germination percentages and consistent early growth responses in previous studies (25 °C being repeatedly reported as ideal for germination across diverse sorghum genotypes) (Patanè et al., 2021; Wang et al., 2025). Controlled chamber photoperiods ensure uniform physiological conditions across accessions, minimizing external variability. Before sowing, seeds were rinsed with distilled water and surface-sterilized briefly with 75% ethanol, followed by rinsing with sterile distilled water. Treated seeds were evenly placed in culture containers lined with filter paper. Two treatments were applied: 1) control (CK), where the seeds were imbibed with distilled water without NaCl, and 2) salt stress, where the seeds were imbibed with distilled water containing 150 mM NaCl. The solution volume was maintained consistently throughout germination, with fresh solution added as necessary to prevent concentration changes due to evaporation. Seed germination and seedling growth were recorded daily until completion.

### 2.3 Germination phenotypic measurement

During germination and at the end of the experiment, germination and seedling growth traits were measured for each sorghum accession under CK and salt stress conditions. All traits were analyzed on a per-accession basis, and the mean values of three biological replicates were used for subsequent analyses.

#### 2.3.1 Germination rate

Germination rate (GR) reflects the final germination ability of seeds over the experimental period (Zhang et al., 2020). Seeds were considered germinated when the radicle emerged through the seed coat. At the end of the germination period, the number of normally germinating seeds was recorded, and GR was calculated as the percentage of germinating seeds relative to the total number of sown seeds:

$GR=\frac{N_{g}}{N_{t}}\times 100$Where N_g_ is the number of normally germinating seeds at the end of the experiment, and N_t_ is the total number of seeds sown.

#### 2.3.2 Germination potential

Germination potential (GP) reflects both the rate and uniformity of seed germination during the early stages of germination (Domin et al., 2019). At a pre-defined early time point (e.g., day d after sowing), the number of germinated seeds was recorded, and GP was calculated as the percentage of germinated seeds relative to the total number of sown seeds:


$$GP=\frac{N_{g,d}}{N_{t}}\times 100$$

Where N_g,d_ is the number of germinated seeds on day d, and N_t_ is the total number of sown seeds.

#### 2.3.3 Germination index

The germination index (GI) provides a comprehensive measure of both the number of germinating seeds and the speed of germination, and describes the dynamic pattern of the germination process (Kader, 2005). GI was calculated by weighting the number of newly germinated seeds at each observation time point:


$$GI=\sum_{i=1}^{k}\frac{G_{i}}{D_{i}}$$

Where G_i_ is the number of newly germinated seeds on day i, D_i_ is the number of days from sowing to day i, and k is the total number of observation days.

#### 2.3.4 Vigor index

The vigor index is used to comprehensively evaluate seedling growth performance during the germination period (Teixeira et al., 2021). It integrates germination dynamics with seedling biomass and was calculated as follows:

*VI = GI × W*

Where *GI* is the germination index, and *W* represents the seedling biomass indicator such as the mean fresh weight or dry weight per seedling).

#### 2.3.5 Determination of fresh and dry weight per plant

At the end of the germination experiment, uniformly growing seedlings were selected for biomass determination. The fresh weight of roots and shoots per plant was measured immediately after gently removing surface moisture with absorption paper. Subsequently, the samples were oven-dried at 70–80 °C to constant weight, and the dry weight of roots and shoots per plant was recorded.

### 2.4 Salt stress retention and overall salt tolerance score calculation

To eliminate the influence of baseline differences among materials, the salt stress retention ( R) for each trait was calculated as:


$$R=\frac{{\text{Trait}}_{NaCl150}}{{\text{Trait}}_{\text{CK}}}$$

where ${\text{Trait}}_{NaCl150}$ and ${\text{Trait}}_{\text{CK}}$ represent the trait values under 150 mM NaCl stress and control conditions, respectively.

The Rvalues of all traits were then standardized using the Z-score method. The overall Salt Tolerance Score (STS) for each accession was obtained by averaging the standardized values of all traits, providing a comprehensive measure of salt tolerance.

### 2.5 qRT-PCR validation of salt stress-related candidate genes

To validate the molecular differences between salt-tolerant and sensitive groups identified by the Salt Tolerance Score (STS), quantitative real-time PCR (qRT-PCR) analysis was performed on 10 representative varieties selected from the extreme STS groups. Five varieties were salt-tolerant (GZ196, GZ349, GZ177, GZ410, GZ471), and five as salt-sensitive (GZ337, GZ233, GZ100, GZ356, GZ238). Samples were taken from each variety under control (CK, 0 mM NaCl) and salt stress treatments (NaCl 150, 150 mM NaCl) to detect transcriptional changes in salt stress-related candidate genes. Eight genes previously reported to be associated with salt tolerance were analyzed, representing functional categories including ion transport, ion homeostasis, and transcriptional regulation. These genes were: *SbNHXLP* (Kumari et al., 2017), *SbHKT1;4* (Wang et al., 2014), *SbHKT1;5* (Guo et al., 2023), *SbCLCc* (Guo et al., 2023), *SbTEF1* (Liu et al., 2024), *SbMYBHv33* (Zheng et al., 2023), *SbWRKY50* (Song et al., 2020), and *SbbHLH85* (Song et al., 2022). Each treatment included three biological replicates, with independent sampling, RNA extraction, and reverse transcription for each replicate. Total RNA was extracted using TRIzol™ Reagent (Invitrogen, USA) following the TRIzol™ Reagent User Guide (Catalog #15596026, Invitrogen, USA). Integrity was assessed by agarose gel electrophoresis, and purity and concentration were evaluated by spectrophotometry. Qualified RNA samples were reverse-transcribed into cDNA using a commercial reverse transcription kit. qRT-PCR reactions were performed in a real-time quantitative PCR system according to the manufacturer’s instructions. Technical replicates were set up for each sample to ensure detection stability. Relative gene expression levels were calculated using the 2^-ΔΔCt^ method, with expression levels under the CK condition served as calibrators (Livak and Schmittgen, 2001). Therefore, relative expression values of each gene under the CK condition were approximately 1. To meet the statistical assumptions, expression data were log^2^ transformed before analysis. Within-accession comparisons between CK and salt stress were performed using paired tests. Between-group differences (salt-tolerant vs. salt-sensitive) under the same treatment conditions were performed using Welch’s t-test. Statistical significance was set at *P < 0.05* (Ruxton). The primers used for all genes in this study are listed in **Supplementary Table 1**.

### 2.6 Root physiological indicators and Na^+^/K^+^ content determination

To verify the differences in oxidative stress response and ion homeostasis between salt-tolerant and sensitive varieties, the representative salt-tolerant variety GZ196 and salt-sensitive variety GZ337 were selected. Root samples were collected under CK (0 mM NaCl) and salt stress (150 mM NaCl) conditions, with three biological replicates (n = 3) per treatment. Samples were quickly rinsed with deionized water, blotted dry, and divided into two portions: one for the physiological indicator and the other for ion content determination. For physiological indicator determination, fresh root tissue was used to prepare extracts according to kit requirements. The contents of malondialdehyde (MDA) (Yu et al., 2026), hydrogen peroxide (H^2^O^2^) (Jiang et al., 2024), superoxide dismutase (SOD) (Cao et al., 2024), and peroxidase (POD) (Liu et al., 2025) activities, and proline (Pro) content (Liu et al., 2023) were determined using a Solarbio kit (Solarbio, Beijing, China). Absorbance readings were taken at the wavelengths specified by the kits, and results were calculated according to the manufacturer’s protocols. For Na^+^/K^+^ content determination, the second portion of roots was oven-dried to constant weight, ground, and sieved. A measured amount of the powder was digested/extracted with acid and brought to a defined volume. The Na^+^ and K^+^ concentrations were determined using flame atomic absorption spectrometry (FAAS) or flame photometry (Oliveira et al., 2010). Ion content was expressed on a dry weight basis, and the K^+^/Na^+^ ratio was calculated. Data are presented as mean ± SD. Differences between GZ196 and GZ337 under the same treatment were analyzed using Welch’s t-test (Ruxton). For comparison within the same accession between CK and salt-stress, paired t-test were performed. Statistical significance was set at *P < 0.05*.

### 2.7 Statistical analysis and multivariate analysis methods

Differences in traits between CK and salt stress treatment for the same accession were analyzed using paired t-tests, with a significance threshold of P < 0.05. To explore multi-trait patterns of salt tolerance, principal component analysis (PCA) and K-means clustering (k = 3) were conducted based on the relative preservation matrix (R matrix) to reveal the distribution of accessions in the multi-trait salt tolerance space (Chen et al., 2024). The stability of the Salt Tolerance Comprehensive Score (STS) ranking was evaluated using bootstrap resampling. In parallel, a Random Forest classification model was constructed to assess the predictive ability of multi-trait phenotypes for salt tolerance levels, with receiver operating characteristic (ROC) curves and area under the curve (AUC) values calculated (Fawcett, 2006; Medina et al., 2020; Tohidi and Olafsson, 2025). Furthermore, canonical correlation analysis (CCA) was used to compare the consistency of phenotypic spaces under CK and salt stress conditions, and the Isolation Forest method was used to identify varieties with anomalous salt stress response patterns (Liu et al., 2012; Winkler et al., 2020).

### 2.8 Software environment and version information

All data processing, statistical analysis, and figure generation were performed in a Python environment. The main software and library versions used were: Python 3.12; pandas 2.x; numpy 1.26; scipy 1.11; scikit-learn 1.4; matplotlib 3.8; joblib 1.3.

## 3 Results

### 3.1 Relative retention of multiple traits reveals salt tolerance cluster structure during germination

The relative retention (R) matrix, constructed from multiple germination and seedling traits, was used to comprehensively assess the responses of 100 sorghum accessions to salt stress. Principal component analysis (PCA) was performed on the standardized R-matrix indicated that the first two principal components captured the main sources of phenotypic variation (**Supplementary Figure 1**). PC1 mainly reflects the overall retention capacity of the key traits, such as germination index, germination rate, and vigor index, while PC2 reflects differences in the relative response among individual traits. In the PCA space, accessions were not randomly distributed but exhibited a clear clustering pattern. K-means clustering analysis (k=3) further divided the accessions into three phenotypic groups: salt-tolerant, intermediate, and sensitive (**Supplementary Figure 1**). Salt-tolerant accessions have higher PC1 scores, indicating that they maintain relative performance under salt stress across most traits. Conversely, salt-sensitive accessions were concentrated in the negative PC1 region, showing pronounced inhibition across multiple traits under salt stress.

**Figure 1:**
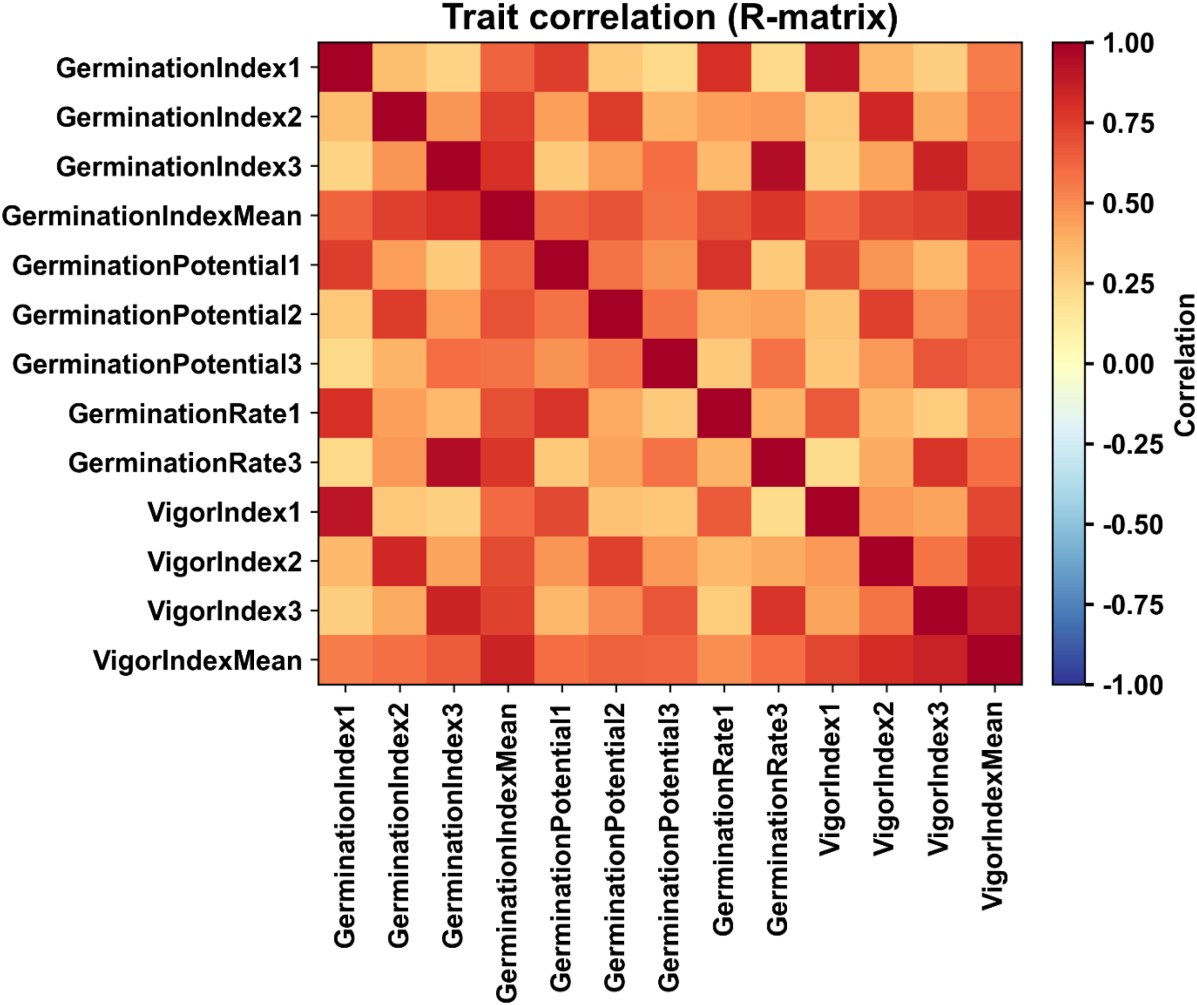
Correlation heatmap of germination and vigor traits under salt stress. **(A)** Germination Index Mean. **(B)** Germination Index1. **(C)** Germination Rate3. **(D)** Germination Index2 . **(E)** Germination Index3. **(F)** Germination Potential3. Color represents Pearson correlation coefficients (0.1 to 1.0), indicating the strength of synergistic retention under salt stress. High correlation revealed functionally similar trait modules, informing trait redundancy and weight allocation in salt tolerance evaluation. The X-axis represents germination and vigor traits, including Germination index, Germination potential, Germination rate, vigor index, whereas the Y-axis represent same traits as the X-axis.

To further analyze the coordinated responses among multiple traits, pairwise correlations were calculated based on the R matrix, and a heatmap was generated (**Figure 1**). The analysis revealed a strong positive correlation among germination index, germination rate, and vigor index, forming a clear correlation module. This indicates that these traits respond synergistically under salt stress. The observed correlation structure provides a statistical basis for subsequent multi-trait integration and the calculation of a comprehensive Salt Tolerance Score (STS).

### 3.2 Significant inhibitory effect of salt stress on key germination traits

**Figure 2:**
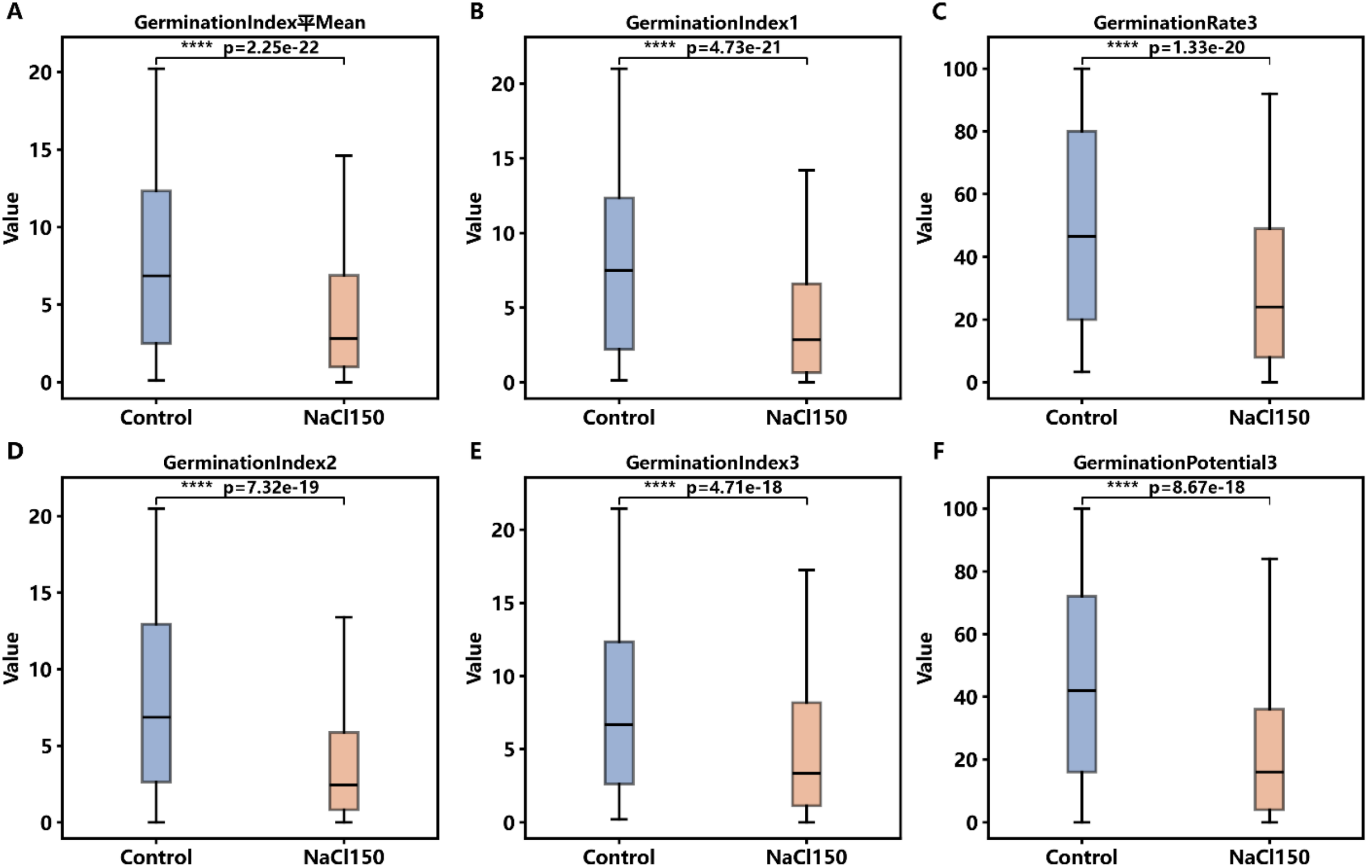
Comparative analysis of germination traits under control and salt stress. Distribution of six germination traits measured under control (CK) and 150 mM NaCl stress. Significance levels from paired tests are shown, demonstrating strong inhibitory effects of salt stress across all traits (p < 1e-17). These phenotypic differences enable the identification of salt-tolerant versus salt-sensitive genotypes. **(A)** Germination Index Mean, **(B)** Germination Index1, **(C)** Germination Rate3, **(D)** Germination Index2, **(E)** Germination Index3, **(F)** Germination Potential3. ns = not significant; * p < 0.05; ** p < 0.01; *** p < 0.001; **** p < 0.0001.

To evaluate the effect of salt stress on sorghum germination, six representative traits with high significance and effect size were selected, and box plots were plotted under CK and 150 mM NaCl treatment (**Figure 2**). Under salt stress, the median values and overall distributions of these traits were lower than under the control condition, and the distribution ranges of most traits were narrowed. Paired statistical tests confirmed that differences between CK and 150 NaCl were all highly significant (p < 0.001). The combined effect size-significance analysis (**Supplementary Figure 3**) showed that the germination index and vigor index had both large effect sizes and extremely high significance, placing them in the “high effect-high significance” region. These results indicate that salt stress has a systematic inhibitory effect on the germination process. The germination index and vigor index, being highly sensitive, may serve as key indicators for assessing salt stress responses during germination.

### 3.3 Consistency of trait ranking under CK and salt stress conditions and cross-stress correlation

Although salt stress significantly reduces the absolute level of measured traits, the relative ranking of accessions across environments is a critical factor in assessing the stability of trait-based screening (Pour-Aboughadareh et al., 2022). Using the top 6 key traits as an example, scatter plots comparing CK and salt stress conditions were generated (**Supplementary Figure 2**). The results showed that most traits exhibited significant positive correlation across the two conditions, indicating that the relative ranking of accessions for these traits remains largely stable under salt stress.

Cross-environment relationships for key germination traits under control (CK) and NaCl150 conditions. Each line corresponds to a different trait metric, with Pearson correlation coefficients (r) and significance levels (p) provided. The strong positive correlations demonstrate that baseline phenotypic performance under control conditions is indicative of salt stress tolerance.

**Figure 3:**
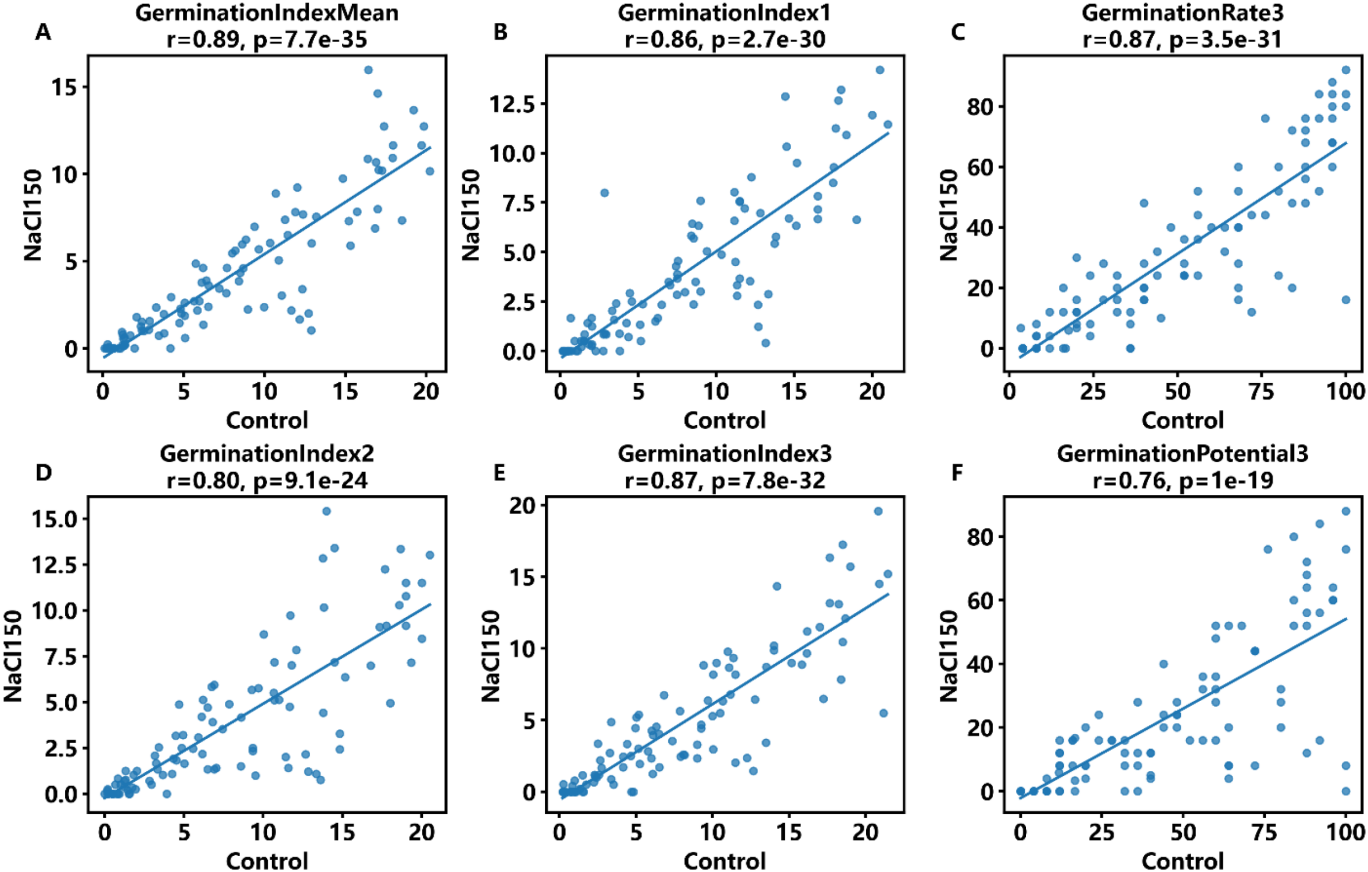
Correlation of phenotypic traits under control and salt stress conditions. **(A)** Germination Index Mean. **(B)** Germination Index1. **(C)** Germination Rate3. **(D)** Germination Index2 . **(E)** Germination Index3. **(F)** Germination Potential3. ns = not significant; * p < 0.05; ** p < 0.01; *** p < 0.001; **** p < 0.0001.

These findings support the use of control (CK) phenotypes for preliminary screening of salt tolerance during the early stages of breeding. At the overall level, genotypic correlation coefficients between CK and NaCl150 conditions were calculated for all traits and visualized as a bar chart (**Figure 3**). The results revealed notable differences in the cross-stress stability among traits. Specifically, the mean germination index, GI3, and certain germination rate traits exhibited high correlations across environments, whereas the stability of other traits was comparatively lower.

### 3.4 Response spectrum analysis and differences in material response patterns

**Figure 4:**
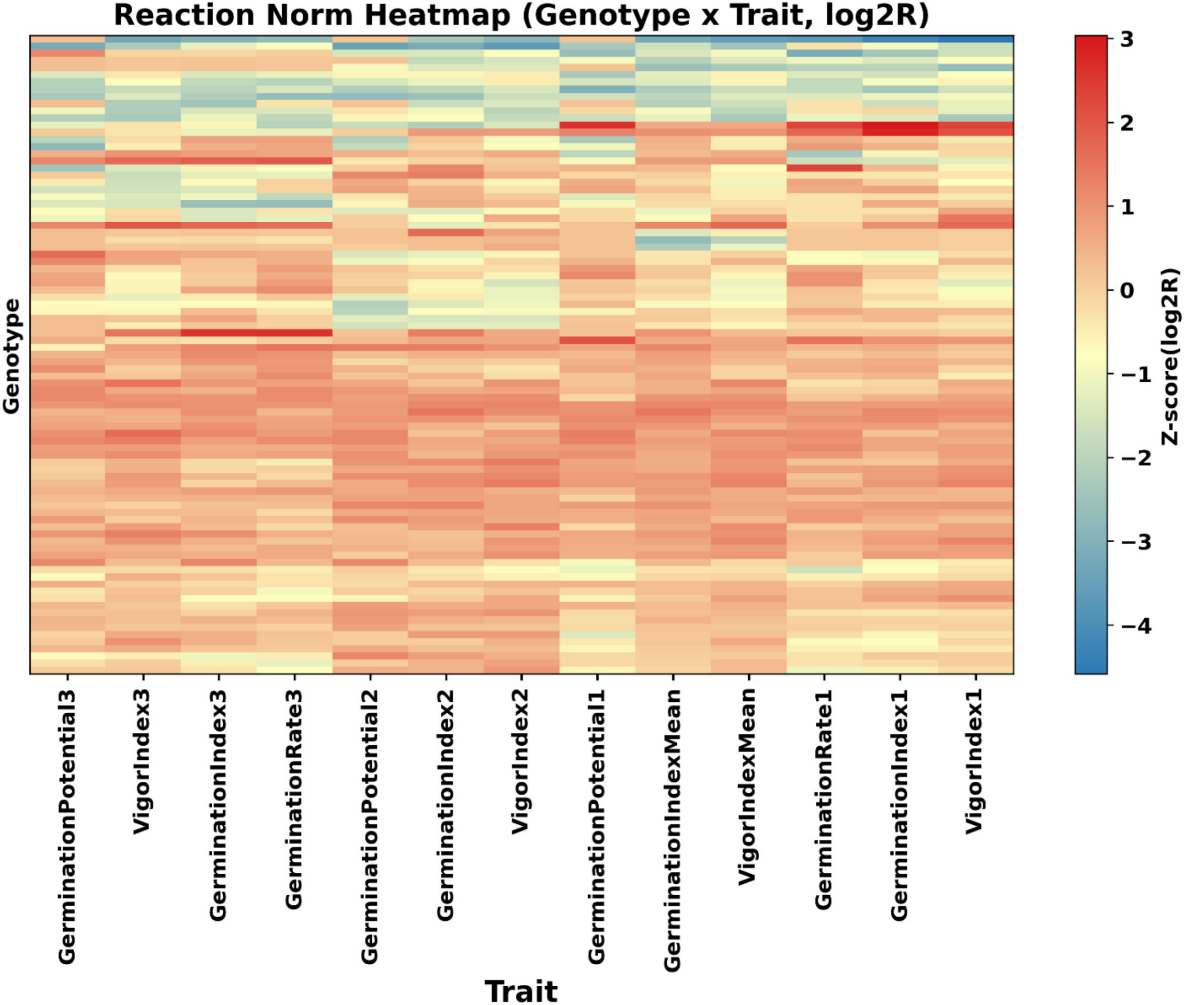
Heatmap of trait response patterns under salt stress (log2R). Colors indicate trait preservation (red) or decline (blue) under salt stress. Hierarchical clustering groups genotypes and traits with similar response patterns, distinguishing broad tolerance from trait-specific adaptations. The X-axis represents 13 wheat genotypes (Vygontides0–3 and Germinalindependents lines), whereas the Y-axis shows the traits (Z-scored log2R values, –4 to 3).

To characterize the direction and intensity of responses across different traits, a response spectrum clustering heatmap was generated based on the log^2^-transformed R matrix (**Figure 4**). The results show that different accessions exhibit clearly distinct response patterns across multiple trait dimensions.

### 3.5 Salt tolerance comprehensive score, material ranking, and stability assessment

The distribution of the Salt Tolerance Scores (STS) across the sorghum population shows a continuous variation in germination performance under salt stress, rather than a simple dichotomy between tolerant and sensitive genotypes (**Supplementary Figure 4**). This reflects the quantitative nature of salt tolerance as a complex trait influenced by multiple phenotypic components.

Ranking materials based on STS enabled the consistent identification of salt-tolerant and sensitive genotypes (**Supplementary Figure 5**). Certain materials consistently exhibited high STS values, while others showed persistently low scores across different indices and repeated analyses, indicating that their salt- tolerant or sensitive characteristics are highly reliable. These rankings highlight representative genotypes at both extremes of the tolerance spectrum, providing clear candidates for further evaluation and potential breeding applications.

**Figure 5:**
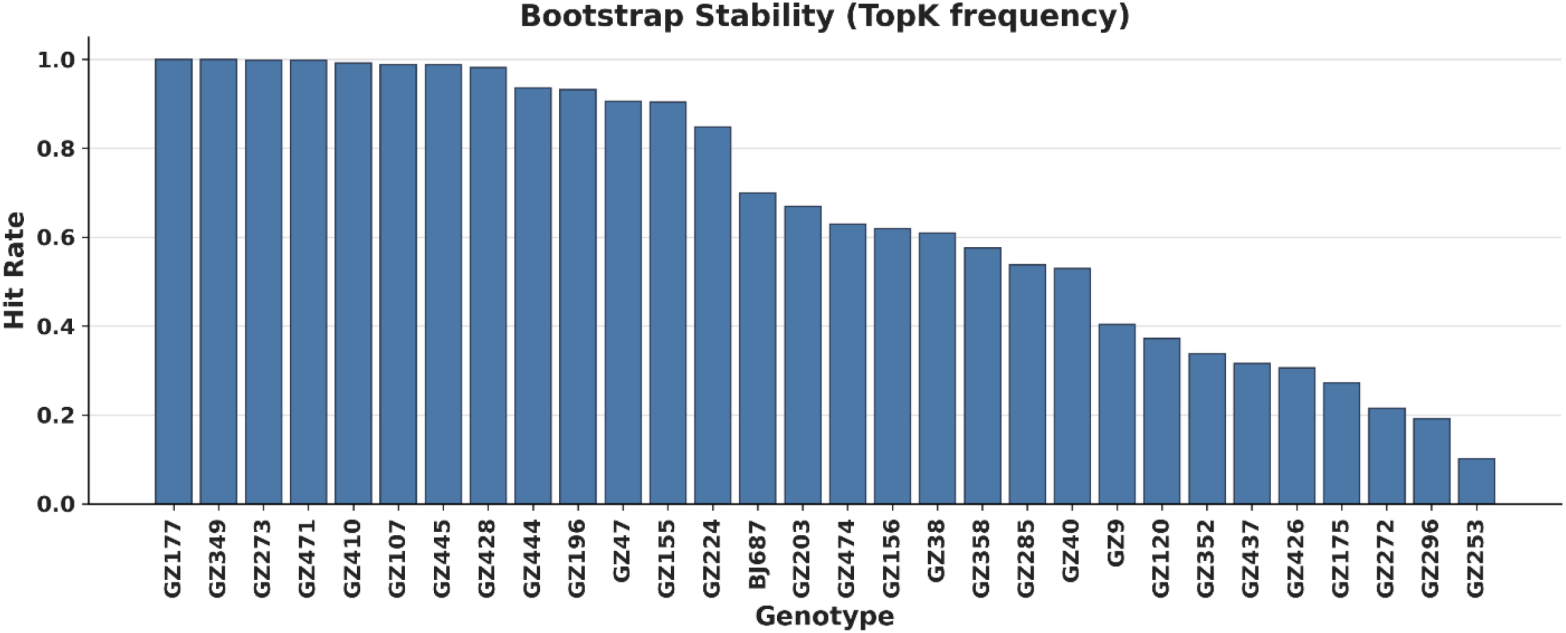
Bootstrap evaluation of salt tolerance ranking stability. The plot illustrates the hit rate (frequency) at which specific materials were ranked within the Top 20 for salt tolerance across multiple bootstrap resampling iterations of the trait dataset. A higher hit rate indicates greater stability of a material’s ranking against trait perturbations, identifying candidates with robust salt tolerance.

To assess the robustness of the STS rankings, Bootstrap resampling was applied (**Figure 5**). The results show that most of the top-performing salt-tolerant materials appeared with high frequency across multiple resampling iterations, confirming the stability of the multi-trait integration approach. This robustness ensures that the identified tolerant and sensitive materials are not artifacts of sample variations, reinforcing their suitability as reliable germplasm resources for breeding programs. Overall, the STS-based ranking framework effectively captures the continuous variation in sorghum salt tolerance, identifies representative extreme genotypes, and demonstrates high stability, providing a solid foundation for downstream applications in germplasm selection and breeding.

### 3.6 Machine learning-assisted salt tolerance typing and key trait identification

To further evaluate the discriminative ability of multi-trait integrated phenotypes, I salt tolerance typing from a data-driven perspective, and to analyze the relative contribution of different traits, we constructed a Random Forest (RF) classification model. This model was based on the Comprehensive Salt Tolerance Score (STS) and its related trait characteristics to distinguish between salt-tolerant and sensitive extreme materials.

#### 3.6.1 Model performance

The ROC curve results show that, under the current extreme material grouping conditions, the Random Forest model effectively distinguishes between salt-tolerant and sensitive materials, with an AUC value of 1.000 (**Supplementary Figure 6**). This indicates a clear separation between the two types of materials in the multi-trait phenotype space. These results demonstrate that the multi-trait features derived from STS can statistically and stably characterize overall differences between salt-tolerant and sensitive materials, providing a strong foundation for subsequent trait contribution analysis.

#### 3.6.2 Trait important analysis

Gini importance analysis based on the Random forest model revealed the relative contributions of different traits in salt tolerance typing (**Supplementary Figure 7**). Traits related to germination rate and early growth vigor (such as germination index and vigor index indicators) had high importance rankings, suggesting that they play a key role in distinguishing between salt-tolerant and sensitive materials. These findings are consistent with the key traits identified from statistical analysis and effect size assessment, indicating that machine learning methods capture biologically meaningful trait importance.

#### 3.6.3 Permutation importance and model robustness

Permutation importance analysis was then used to evaluate the impact of single trait perturbations on the model’s discriminative performance (**Supplementary Figure 8**). Most traits had permutation importance values close to zero, with only a few showing slight performance decreases. This suggests that the Random Forest model’s discriminative ability does not rely on any single trait but arises from the synergistic effect of multiple highly correlated traits. Based on correlation analysis, the multi-trait integration framework exhibits both high redundancy and robustness, making the model resilient to perturbations in individual traits.

Finally, using the upper and lower quantiles of the STS (Sodium Salt Tolerance Scale) as salt-tolerant and sensitive categories, the Random Forest model was applied to R-matrix phenotypic features. The ROC curves and AUC values confirmed that multiple phenotypic features effectively predict the salt tolerance levels of materials. Ranking traits by Gini importance further highlighted key phenotypic indicators that dominate overall salt tolerance discrimination.

Overall, Random Forest analysis verifies the effectiveness of the multi-trait integration framework in salt tolerance typing, demonstrating both strong overall discriminative ability and meaningful trait contributions. Salt tolerance emerges not from a single index but from the combined effect of multiple germination and early growth traits.

### 3.7 Overall association between CK and salt stress phenotypic space and identification of anomalous response materials

**Figure 6:**
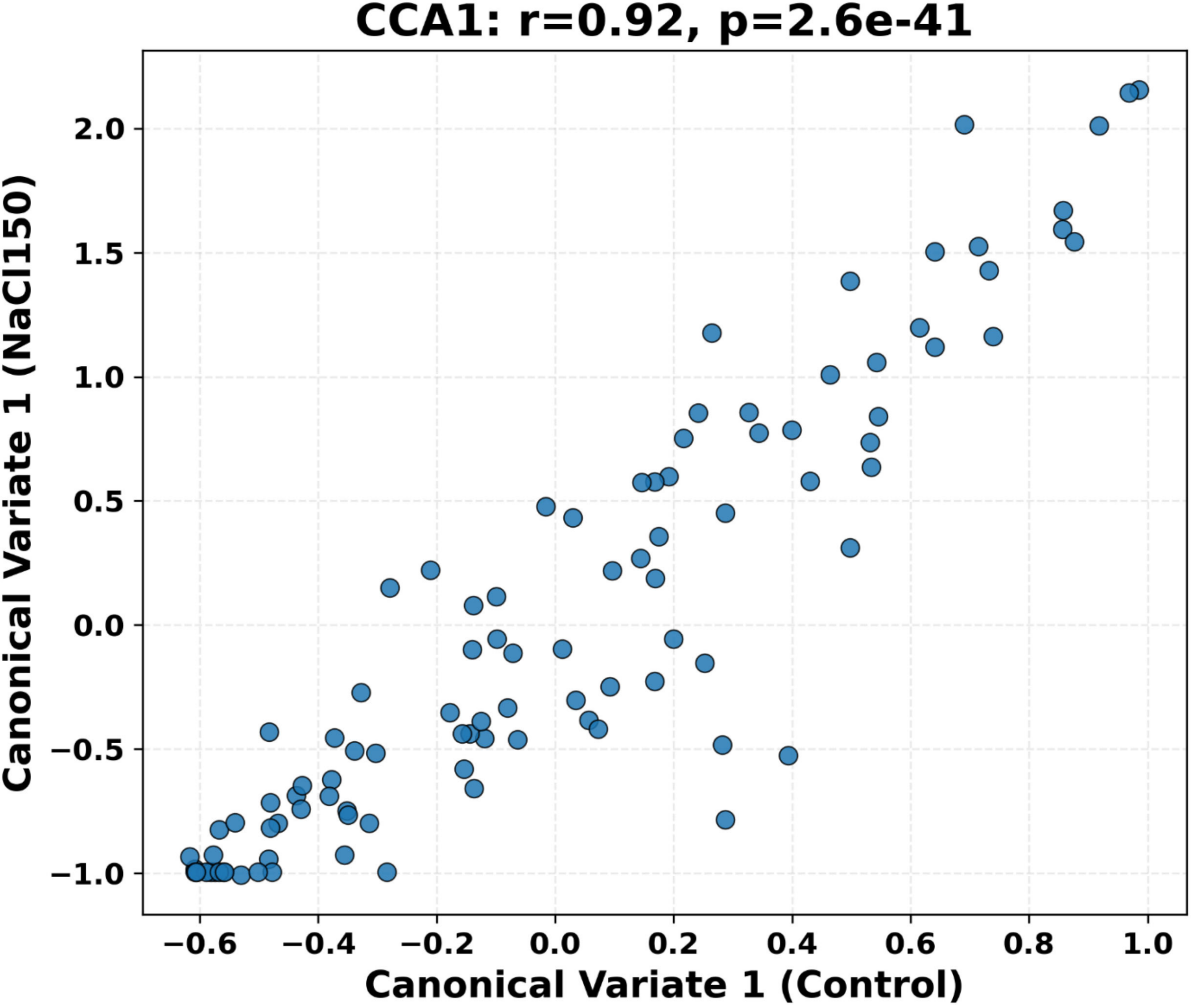
Canonical correlation analysis of phenotypic spatial consistency between CK and salt-stress conditions. The scatter plot shows the distribution of canonical variate 1 (CCA1) scores, reflecting the structural alignment of phenotypes across the two environments. This supports the potential of using baseline phenotypic data to predict salt stress responses.

Canonical correlation analysis (CCA) was used to assess the overall association between the CK and the multi-trait phenotypic space under salt stress conditions. The results revealed a significant correlation between the two environments, indicating that although salt stress significantly alters absolute trait values, the overall phenotypic structure retains notable commonalities (**Figure 6**).

Furthermore, the Isolation Forest algorithm was used to identify anomalous response accessions in log^2^R space, and their distribution in PCA space is shown in (**Supplementary Figure 9**). These accessions significantly deviate from the main population in multi-trait response patterns, possibly representing specific salt-tolerant or sensitive response types, and are worthy of further validation and in-depth research.

### 3.8 Transcriptional response differences of eight salt stress-related genes in extreme materials

**Figure 7:**
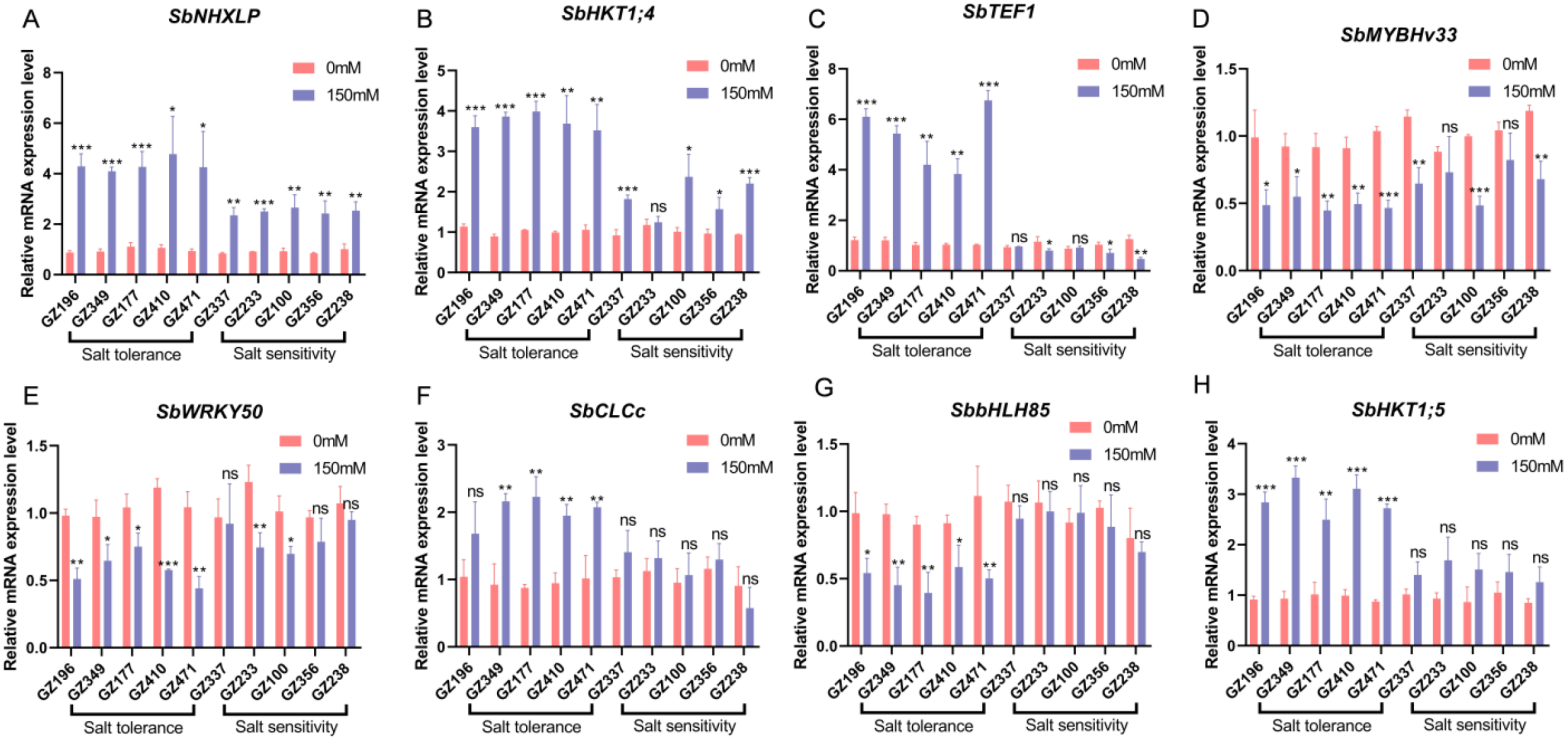
Expression patterns of eight salt stress-related genes in salt-tolerant and salt-sensitive sorghum accessions under control and salt stress conditions. Relative mRNA expression levels of eight previously reported salt stress-related genes in ten sorghum accessions under control (0 mM NaCl) and salt stress (150 mM NaCl) conditions. Five accessions represent salt-tolerant genotypes (GZ196, GZ349, GZ177, GZ410, and GZ471), and five represent salt-sensitive genotypes (GZ337, GZ233, GZ100, GZ356, and GZ238). Panels represent the expression patterns of different genes: **(A)** SbNHXLP, **(B)** SbHKT1;4, **(C)** SbTEF1, **(D)** SbMYBHv33, **(E)** SbWRKY50, **(F)** SbCLCc, **(G)** SbbHLH85, and **(H)** SbHKT1;5. Bars represent mean ± SD of three biological replicates (n = 3). Red bars indicate the control treatment (0 mM NaCl), and blue bars indicate the salt stress treatment (150 mM NaCl). Statistical significance between treatments was determined using Welch’s t-test. ns, not significant; * p < 0.05; ** p < 0.01; *** p < 0.001.

To further verify the molecular-level differences between salt-tolerant and sensitive accessions identified by the Comprehensive Multi-Trace Score (STS), five representative accessions were selected from each extreme of the STS distribution (salt-tolerant: GZ196, GZ349, GZ177, GZ410, GZ471; sensitive: GZ337, GZ233, GZ100, GZ356, GZ238). Under control (CK) and 150 mM NaCl treatment conditions, qRT-PCR was performed to quantify the expression of eight previously reported salt stress-related genes (**Figure 7**).

Under CK conditions, the relative expression levels of all genes were generally close to 1, showing only slight inter-accession fluctuations, consistent with the normalization against CK values for each accession (**Figures 7A–H**). In contrast, under 150 mM NaCl stress, genes representing different functional categories displayed markedly distinct expression patterns between salt-tolerant and salt-sensitive accessions. For instance, ion homeostasis and transport-related genes were strongly enhanced in salt-tolerant accessions. Specifically, genes involved in Na^+^ compartmentalization and transport, *SbNHXLP* (**Figure 7A**), *SbHKT1;4* (**Figure 7B**), and *SbHKT1;5* (**Figure 7H**) were all significantly upregulated under NaCl 150 treatment, with the induction level markedly higher in salt-tolerant than in sensitive accessions. In salt-tolerant accessions, the relative expression levels of these genes were generally 2–4 fold or higher, while in sensitive accessions, induction was weak or moderate. These results indicate that salt-tolerant accessions maintain ion homeostasis under salt stress by enhancing Na^+^ transport, compartmentalization, and redistribution pathways.

**Figure 8:**
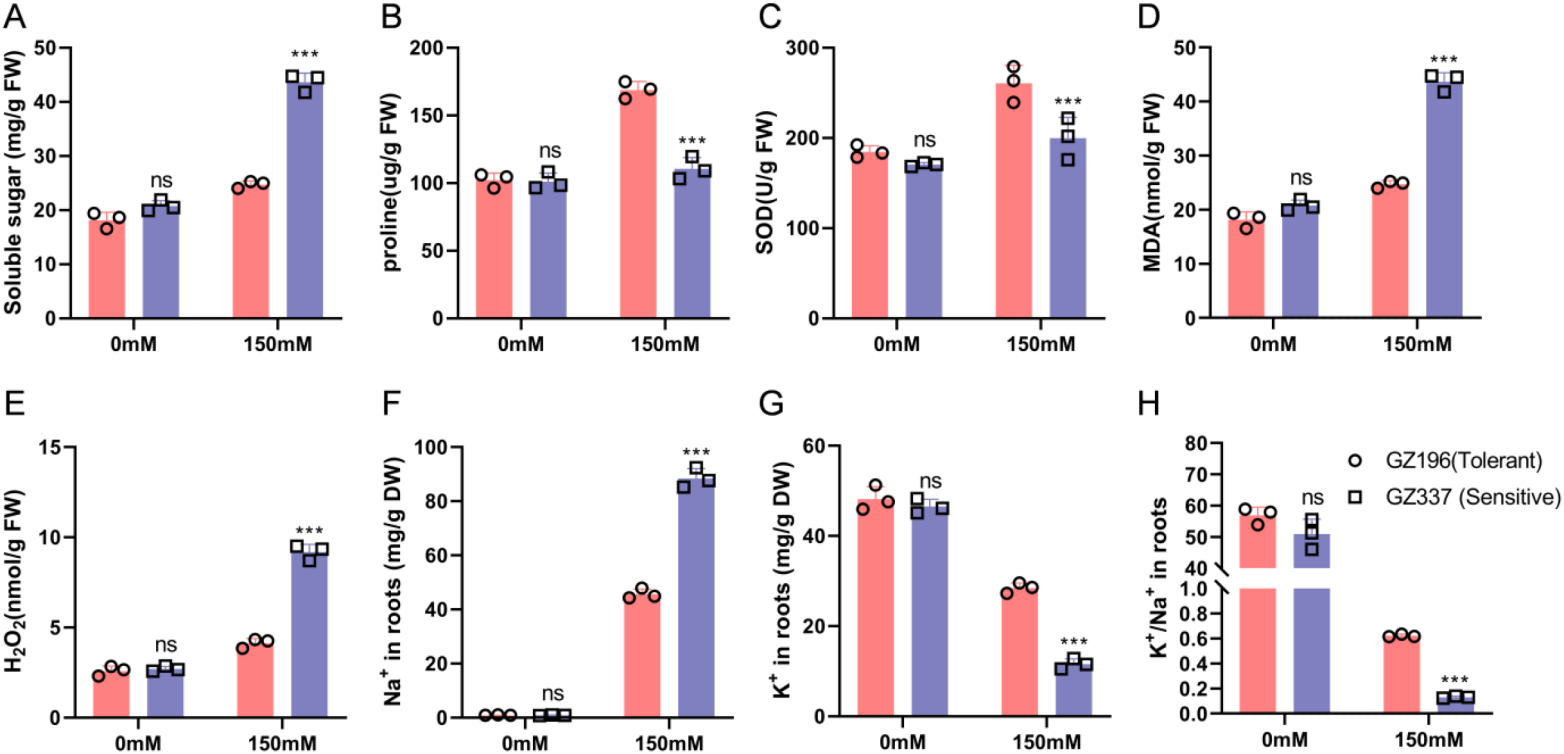
Physiological responses and root ion homeostasis of salt-tolerant accession (GZ196) and a salt-sensitive accession (GZ337) under control and salt stress conditions. **(A)** Malondialdehyde (MDA) content; **(B)** peroxidase (POD) activity; **(C)** superoxide dismutase (SOD) activity; **(D)** proline (Pro) content; **(E)** hydrogen peroxide (H^2^O^2^) content; **(F)** Na^+^ content in roots; **(G)** K^+^ content in roots; **(H)** K^+^/Na^+^ ratio. Bars represent mean ± SD of three biological replicates (n = 3). Red bars indicate the salt-tolerant accession (GZ196), and blue bars indicate the salt-sensitive accession (GZ337). Statistical significance between GZ196 and GZ337 under the same treatment was determined using Welch’s t-test. Different letters or asterisks indicate significant differences (ns = not significant; *** p < 0.001).

Similarly, the Cl^-^ transport-related gene *SbCLCc* (**Figure 7H**) showed moderate upregulation under salt stress, with expression in salt-tolerant accessions generally higher than that in sensitive accessions. However, the induction level was lower than that observed for Na^+^ transport-related genes, suggesting that Cl^-^ compartmentalization functions as an auxiliary mechanism in regulating ion homeostasis. Core response genes also displayed high expression levels in salt-tolerant accessions. For example, *SbTEF1* (**Figure 7C**) was significantly upregulated under NaCl 150 conditions, while it was low-expressed or slightly inhibited in the sensitive accession. This pattern indicates that SbTEF1 may contribute to the stable operation of key transcriptional and metabolic processes under salt stress, representing an important molecular feature distinguishing salt-tolerant from sensitive accessions. In contrast, transcription regulation-related genes showed a downregulation trend in salt-tolerant accessions. *SbMYBHv33* (**Figure 7D**), *SbWRKY50* (**Figure 7E**), and *SbbHLH85* (**Figure 7G**) showed an overall downregulation trend under salt stress conditions, with expression in salt-tolerant accessions significantly lower than in sensitive ones. Notably, *SbbHLH85* maintained consistently low expression in salt-tolerant accessions, while expression was relatively high in sensitive accessions, suggesting a potential role as a negative regulator of the salt stress response. Comprehensive analysis (**Figure 7**) shows that salt-tolerant accessions exhibit a transcriptional response characterized by “significant induction of ion homeostasis-related genes + high expression of key response genes + downregulation of some transcription regulators”, while sensitive accessions showed weaker or opposite responses in these pathways. These molecular-level difference corroborates the phenotypic and physiological results (**Figure 8**), further supporting the reliability of screening salt-resistant accessions using the comprehensive multi-trait STS approach.

The bar chart represents the mean ± standard deviation (n = 3) of three biological replicates. Red bars represent salt-tolerant materials, and blue bars represent sensitive materials. The differences between the salt-tolerant group and the sensitive group under NaCl 150 conditions were statistically analyzed using Welch’s t test, with asterisks indicating statistical significance (P < 0.05; P < 0.01).

### 3.9 Differences in physiological and biochemical responses and root ion homeostasis reveal the salt tolerance mechanism

To investigate the physiological basis of salt tolerance, the salt-tolerant accession GZ196 and the sensitive accession GZ337 were selected, and osmotic regulation and oxidative stress-related indicators were measured under CK and salt stress conditions. Root Na^+^/K^+^ content, as well K^+^/Na^+^ ratio, were also quantified (**Figures 8A–H**); corresponding indicators: **Figure 8A**, MDA; **Figure 8B**, POD activity; **Figure 8C**, SOD activity; **Figures 8D, proline** content; **Figure 8E**, H^2^O^2^ content; **Figure 8F**, Na^+^ content; **Figure 8G**, K^+^ content; **Figure 8H**, K^+^/Na^+^ ratio). Under salt stress, both accessions exhibited significant physiological changes, but the response patterns were distinctly different. For oxidative damage indicators, under salt stress conditions, the MDA (**Figure 8A**) and H^2^O^2^ (**Figure 8E**) of the sensitive accession increased significantly, and both were significantly higher than those of the salt-tolerant accession, indicating greater membrane lipid peroxidation and reactive oxygen species accumulation. In contrast, the salt-tolerant accession exhibited a stronger antioxidant enzyme response under salt stress treatment, with POD (**Figure 8B**) and SOD (**Figure 8C**) activities significantly higher than those of the sensitive accession. This suggests that salt-tolerant accessions can more effectively scavenge ROS and mitigate oxidative stress. Regarding osmotic regulation, proline accumulation (**Figure 8D**) in the salt-tolerant accession under NaCl150 was markedly higher than in the sensitive accession, indicating that the tolerant accession maintains cell water balance and homeostasis by enhancing osmolyte accumulation.

Ion homeostasis results further support the above conclusions. Following salt treatment, Na^+^ content in the roots of the sensitive accession was significantly higher than that in the salt-tolerant accession (**Figure 8F**), while K^+^ content (**Figure 8G**) was significantly lower, resulting in a marked reduction of the K^+^/Na^+^ ratio (**Figure 8H**). Conversely, the salt-tolerant accession effectively limited excessive Na^+^ accumulation and maintained a higher K^+^ level, thereby sustaining a significantly higher K^+^/Na^+^ ratio under salt stress. Overall, these results (**Figure 8**) indicate that salt-tolerant accessions cope with salt stress through a combination of enhanced antioxidant defenses, improved osmotic regulation, and maintenance of K^+^/Na^+^ homeostasis. These physiological adaptations are consistent with the transcriptional upregulation of ion homeostasis–related genes observed in **Figure 7**, providing integrated molecular and physiological evidence for salt tolerance mechanisms.

## 4 Discussion

This study focused on the germination stage of sorghum and constructed a comprehensive salt tolerance evaluation system based on the relative retention of multiple traits, enabling robust differentiation between salt-tolerant and salt-sensitive accessions at the population level. Based on this, the response characteristics of salt-tolerant accessions under salt stress were further validated through molecular and physiological analyses of extreme materials. Overall, this study provides an integrated, multi-level framework for salt tolerance analysis and screening, encompassing phenotypic integration, genotypic stability, molecular response, and physiological mechanisms.

### 4.1 Multi-trait integration reveals continuous variation in salt tolerance

Salt tolerance during germination is not determined by a single trait, but rather by the comprehensive result of the synergistic changes of multiple germination and early growth traits under salt stress (Munns and Tester, 2008). Traditional studies often rely on single indicators such as germination rate or germination potential for screening, which is easily affected by differences in baseline growth potential or measurement fluctuations, thus reducing the stability and transferability of screening results (Song et al., 2025). This study introduced the relative retention index (R), relative decline rate (RD), and response spectrum index (log^2^R), and developed a comprehensive Salt Tolerance Score (STS) based on these indicators. This approach effectively minimized the influence of inherent growth differences under control conditions, enabling the evaluation to more accurately reflect the intrinsic response capacity of accessions to salt stress.

Principal component analysis, cluster analysis, and stability assessment results based on STS showed that salt tolerance during sorghum germination exhibited a continuous distribution characteristic in the population, rather than a simple dichotomy between salt tolerance and sensitivity. This observation aligns with the quantitative nature of salt tolerance and highlights that integrating multiple traits provides a more comprehensive characterization of the salt tolerance response. Furthermore, when combined with a machine learning classification model, STS and its associated traits demonstrated strong discriminative ability for distinguishing between salt-tolerant and sensitive accessions, providing a reliable quantitative tool for early salt-tolerance screening (Liu et al., 2025).

### 4.2 Consistency between phenotypic and molecular response

Following population-scale phenotyping, STS extreme accessions were selected for qRT-PCR validation to assess the molecular basis of the phenotypic screening results. As shown in **Figure 7**, salt stress–related genes representing different functional categories exhibited consistent and biologically meaningful expression differences between salt-tolerant and sensitive accessions. Ion homeostasis and transport-related genes (*SbNHXLP*, *SbHKT1;4*, *SbHKT1;5*, and *SbCLCc*) were generally upregulated under salt stress, with the induction level in the salt-tolerant accession being significantly higher than that in the sensitive one. These results indicate that salt-tolerant accessions tend to alleviate ion toxicity by enhancing processes such as Na^+^ isolation, transport, and Cl^-^ partitioning, thereby maintaining cellular homeostasis during germination and early growth stages (Munns and Tester, 2008; Cui et al., 2020; Liu et al., 2023). Notably, the magnitude of gene induction varied among ion pathways, with Na^+^ transport-related genes generally higher than that of Cl^-^ transport-related genes, suggesting differential regulation of ion-specific stress responses during germination.

Meanwhile, the core response gene *SbTEF1* was significantly upregulated in salt-tolerant accessions, while its expression remained low in the sensitive accession. This phenomenon suggests that salt-tolerant accessions may possess stronger transcriptional and metabolic stability under salt stress, supporting the maintenance of essential physiological processes required for germination and early seedling growth (Wang et al., 2023; Guo et al., 2025).

### 4.3 Differential regulation of transcription factors under salt stress

In contrast to genes related to ion homeostasis, transcriptional regulatory genes such as *SbMYBHv33*, *SbWRKY50*, and *SbbHLH85* showed an overall downregulation under salt stress, with their expression levels in salt-tolerant accessions generally lower than those in sensitive ones. This pattern indicates that, during germination under salt stress, salt-tolerant accessions do not rely solely on sustained activation of the transcriptional regulator. Instead, they may achieve more efficient stress adaptation by completing homeostasis reconstruction earlier or by reducing the need for continuous activation of certain negative regulatory factors.

Notably, the persistently low expression of *SbbHLH85* in salt-tolerant accessions is consistent with its reported role as a negative regulator of salt tolerance, suggesting that this gene may participate in the salt stress response by affecting root structure or ion uptake processes (Song et al., 2022). It is important to emphasize that the effects of these transcription factors are highly time- and context-dependent; their expression changes should not be interpreted simply as “high” or “low”, but considered in conjunction with downstream pathway activity and the overall physiological status of the plant.

### 4.4 Coordinated molecular and physiological mechanisms of salt tolerance

The observed differences in molecular expression were further corroborated at the physiological level. Root ion assays showed that salt-tolerant accessions effectively limited excessive Na^+^ accumulation and maintained high K^+^ levels and K^+^/Na^+^ ratios under salt stress. Simultaneously, their antioxidant enzyme activity and osmotic regulation capabilities were significantly superior to those of sensitive materials. These physiological characteristics corroborate the stronger induction of ion homeostasis-related genes in (**Figure 7**), indicating that salt-tolerant accessions exhibit a synergistic response pattern of “ion homeostasis maintenance, oxidative damage mitigation, enhanced osmotic regulation” under salt stress.

Overall, salt-tolerant accessions achieve adaptation to salt stress not through a single pathway or gene, but via coordinated multi-level regulatory mechanisms. This observation further supports the effectiveness of the STS evaluation system based on multi-trait integration in capturing the complex yet stable response patterns underlying salt tolerance (An et al., 2025; Jin et al., 2025).

### 4.5 Breeding implications and study limitations

From a breeding application perspective, the multi-trait integration evaluation framework proposed in this study is suitable for the rapid screening of salt-tolerant materials during sorghum germination, especially for pre-screening of early-generation populations or large-scale germplasm resources. Combining STS (Salt Tolerance Score) with molecular marker expression profiles can further enhance the interpretability and accuracy of salt tolerance evaluations. It should be noted that this study mainly focused on a single salt stress intensity (150 mM NaCl) during germination. Response patterns of accessions may differ under different salt concentrations or exposure durations. Moreover, while the observed expression differences of candidate genes provide important insights into potential salt tolerance mechanisms, they do not establish direct causal relationships. Further in-depth studies, including genetic mapping, allelic variation analysis, or functional validation, are required to clarify the underlying mechanisms.

## 5 Conclusion

This study established a multi-trait integrated evaluation of farmwork to assess salt tolerance in sorghum during germination based on the relative maintenance of key germination and early growth traits under 150 mM NaCl stress. The constructed Salt Tolerance Score (STS) effectively minimized the influence of baseline growth differences and enabled stable differentiation of salt-tolerant and salt-sensitive materials among 100 sorghum accessions. Molecular and physiological validation of extreme STA materials further supported the reliability of the phenotypic screening results. Salt-tolerant materials exhibited stronger induction of ion homeostasis-related genes, maintained higher K^+^/Na^+^ ratios, enhanced antioxidant defense, and improved osmotic regulation capacity under salt stress. These coordinated responses collectively contributed to superior performance during germination. Overall, salt tolerance in sorghum at the germination stage is determined by multi-trait synergistic maintenance and integrated ion homeostasis regulation rather than by a single trait or gene. The proposed multi-trait integration farmwork provides a robust and practical tool for early-stage salt tolerance screening and breeding applications.

## Data Availability

The datasets presented in this study can be found in online repositories. The names of the repository/repositories and accession number(s) can be found in the article/**Supplementary Material**.
