# Supplementary material for: Multilevel analysis of salt stress responses in sorghum during seed germination
Source: Front Plant Sci. 2026 Mar 19;17:1802398. doi: 10.3389/fpls.2026.1802398 (PMC13044168; doi:10.3389/fpls.2026.1802398)
Supplement: Supplementary file 1 [file Table1.docx]

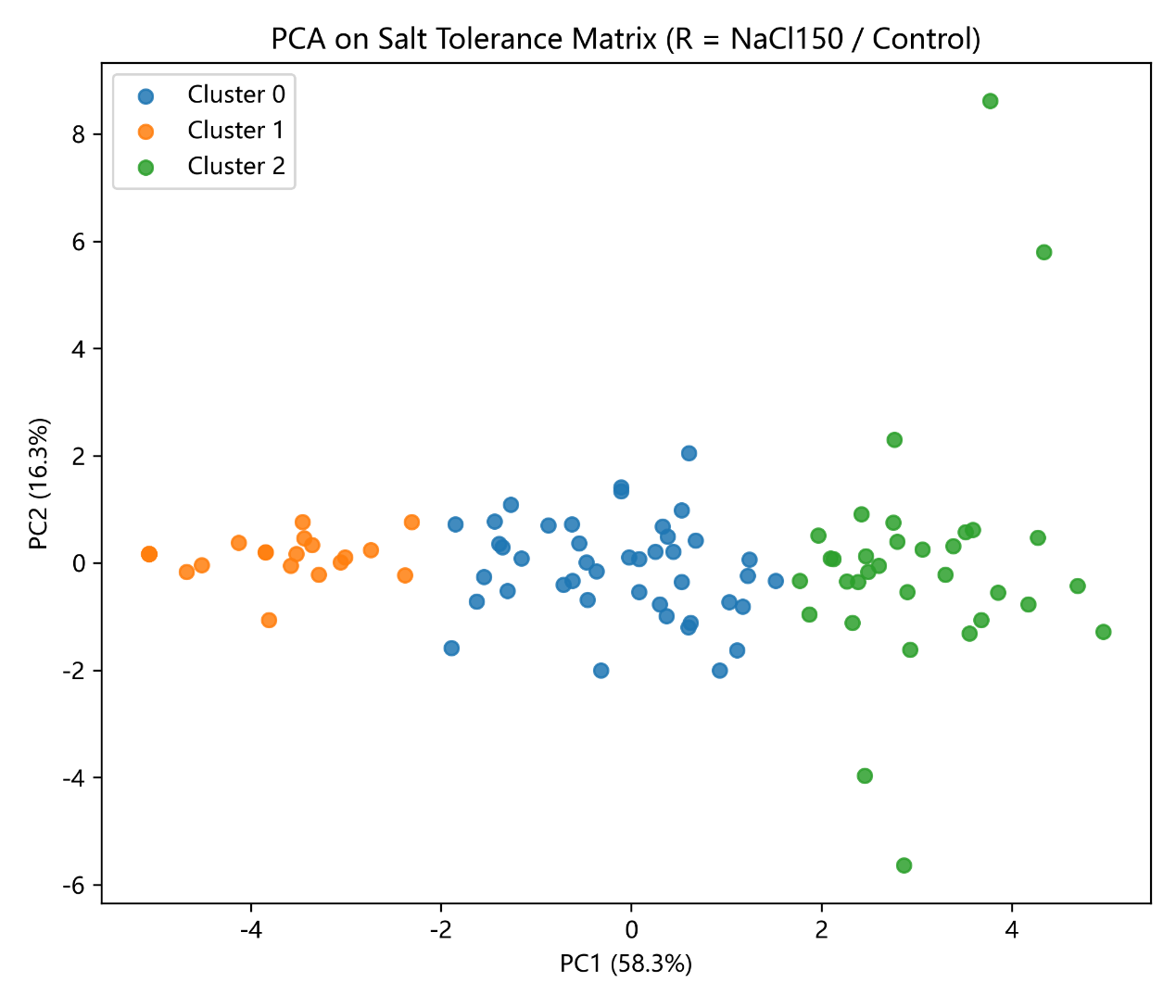


**Fig. S1** | Principal Component Analysis and Material Clustering Based on Relative Retention Matrix.

An R matrix was constructed based on the relative retention of each trait under NaCl150 and CK conditions (R = NaCl150/CK), and principal component analysis (PCA) was performed. Scatter points represent the distribution of different sorghum materials in PC1 and PC2 spaces, and different colors represent K-means clustering results (k = 3). This figure reveals the differentiation pattern of materials in the comprehensive salt tolerance phenotypic space, providing an overall structural reference for subsequent salt tolerance clustering and screening.


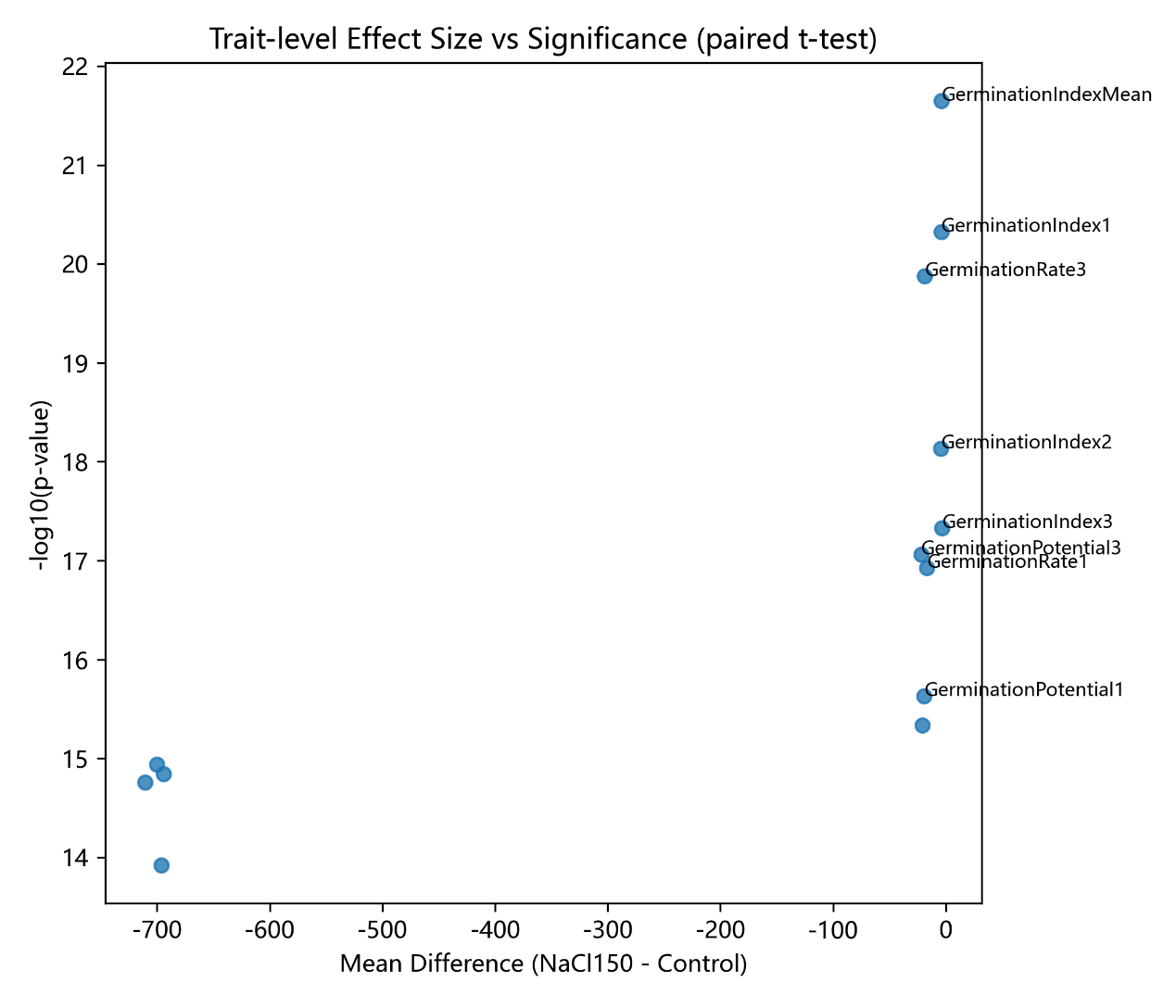


**Fig. S2** | Control of Key Traits under CK and NaCl 150 Conditions – Stress Response Relationship

The paired response relationships of six key traits under CK and NaCl 150 conditions are shown in scatter plot form. Each point represents a single material, and the position of the point relative to the diagonal reflects the strength of trait retention under salt stress. This plot is used to identify material types that are stable or highly sensitive at multiple trait levels, providing a visual reference for screening strategies. From a breeding screening perspective, these traits not only respond strongly to salt stress but also have high weight in both statistical and biological significance, making them the most discriminative core indicator in evaluating salt tolerance during germination.


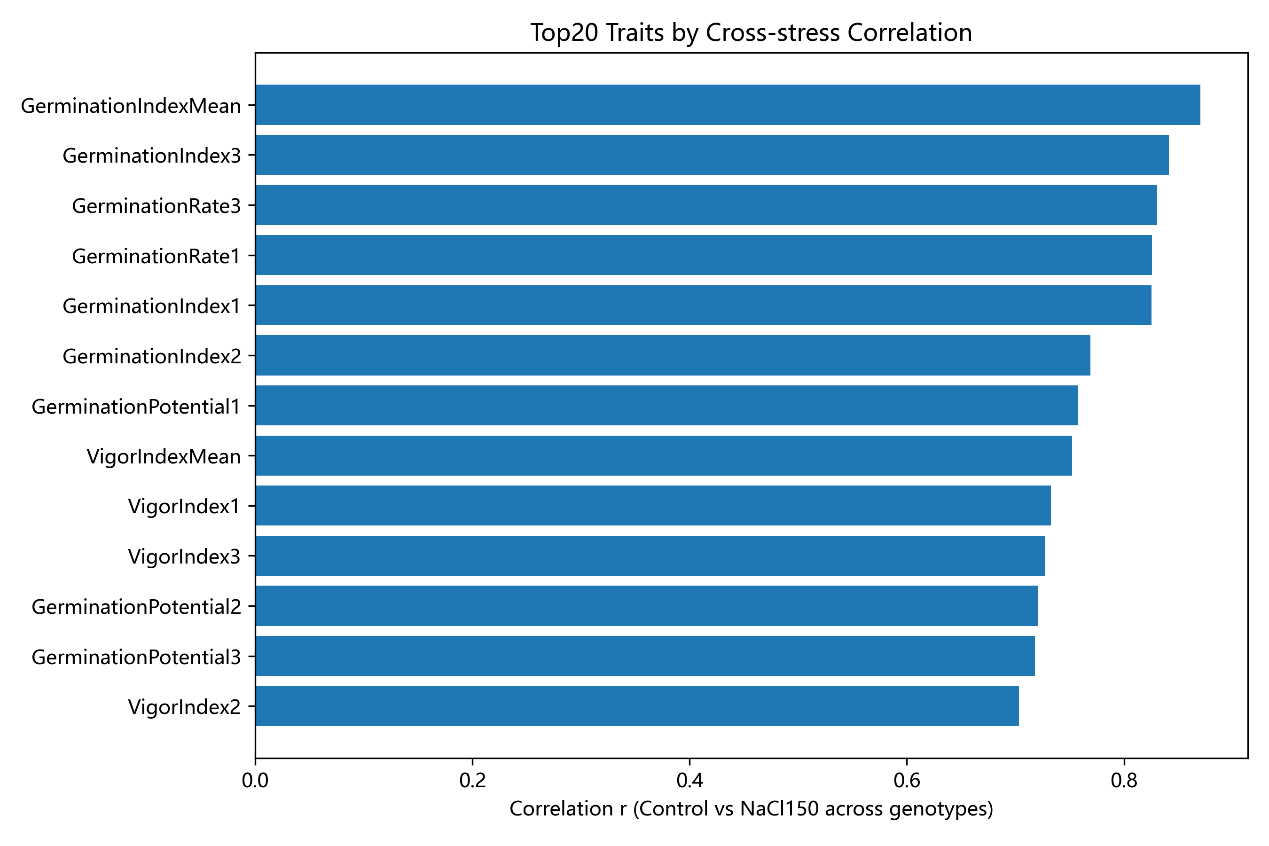


**Fig. S3** | Comprehensive Analysis of Trait Response Effect Size and Significance (Volcano Plot)

The volcano plot simultaneously displays the response effect size and statistical significance level of key traits under salt stress. The horizontal axis represents the magnitude of trait variation, and the vertical axis represents the significance of differences. This plot is used to identify key salt-tolerance related traits that have both large response magnitudes and statistical reliability, providing a basis for constructing an evaluation index system. These results indicate that traits with high cross-stress correlation are more suitable as stable salt tolerance screening indicators, while traits with lower consistency are more suitable for characterizing the response magnitude of salt stress.


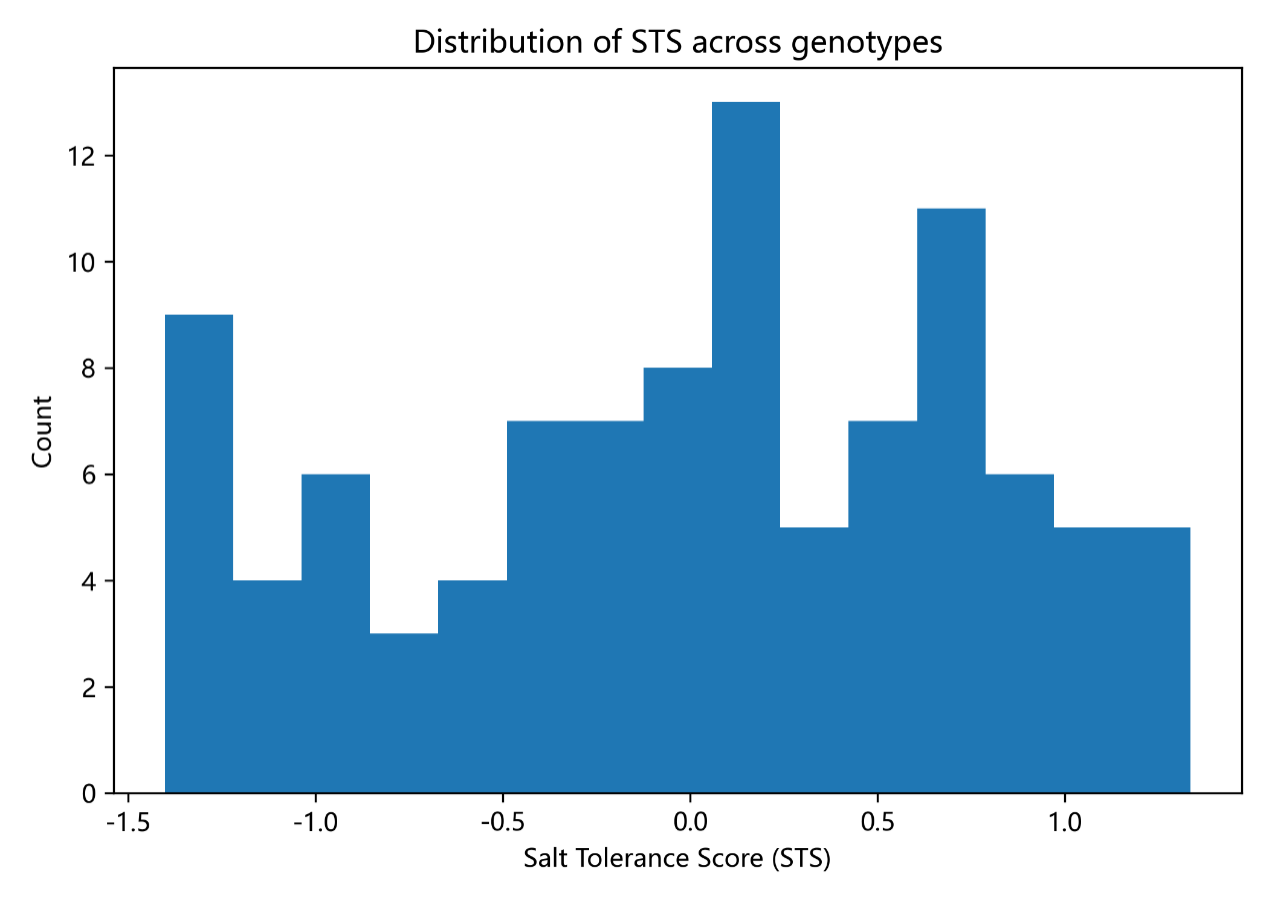


**Fig. S4** | Distribution Characteristics of Salt Stress Tolerance Score (STS)

The Salt Stress Tolerance Score (STS) was calculated based on the standardized relative retention of multiple key traits, and its distribution in the population is shown. STS reflects the continuous variation characteristics of a material's overall salt tolerance. This result provides a quantitative basis for subsequent screening of salt-tolerant materials by threshold or quantile. After ranking by STS, salt-tolerant and sensitive materials can be stably identified (Fig. 9). Some materials consistently exhibited high or low STS across different analytical indices and repeated analyses, indicating that their salt-tolerant or sensitive characteristics are highly reliable.


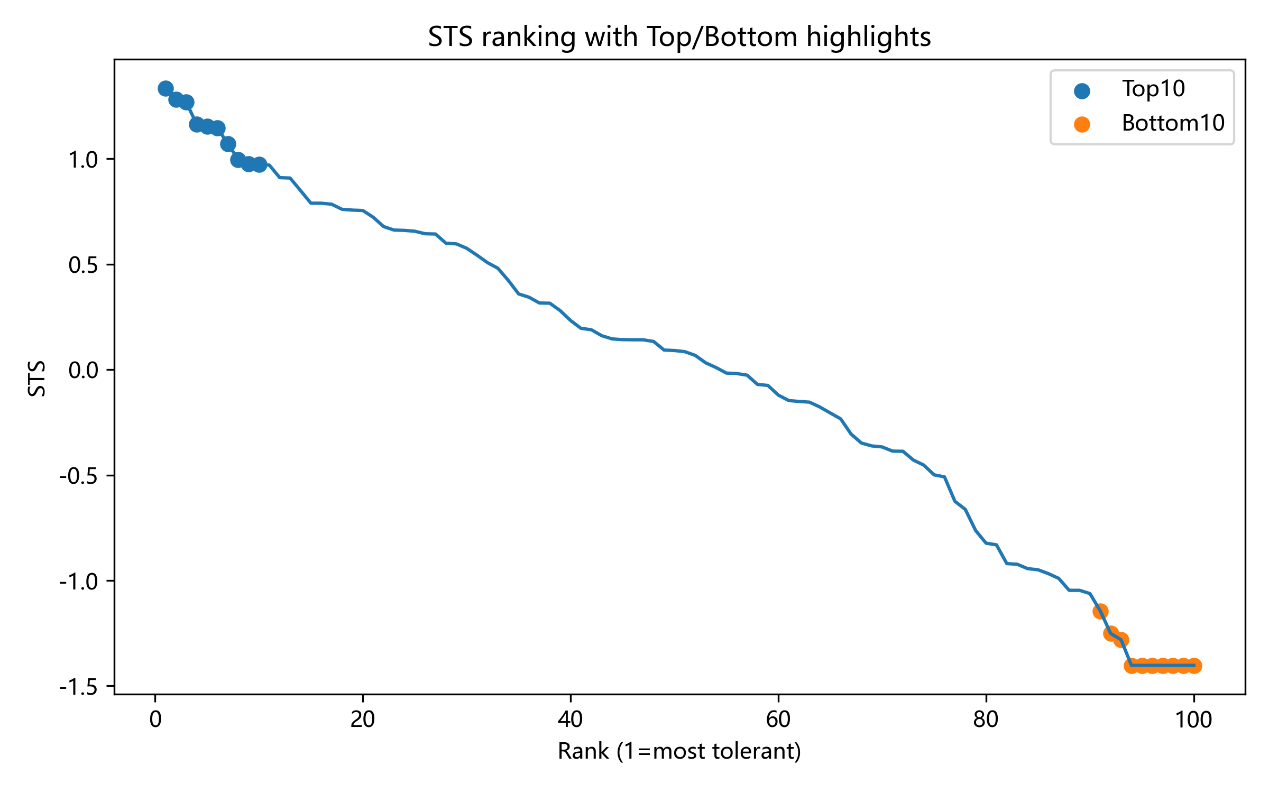


**Fig. 5** | Salt-tolerant material ranking results based on STS (Top and Bottom)

Materials are ranked according to STS, showing representative materials with strong and weak salt tolerance. This ranking is used to visually identify potential salt-tolerant germplasm resources and provide candidates for subsequent stability analysis and breeding applications. Furthermore, the robustness of the STS ranking was evaluated using the Bootstrap resampling method (Fig. 10). The results show that most of the top salt-tolerant materials maintained a high frequency of occurrence in multiple resamplings, indicating that the salt tolerance screening results based on multi-trait integration have good stability and are suitable for material selection in breeding practice.


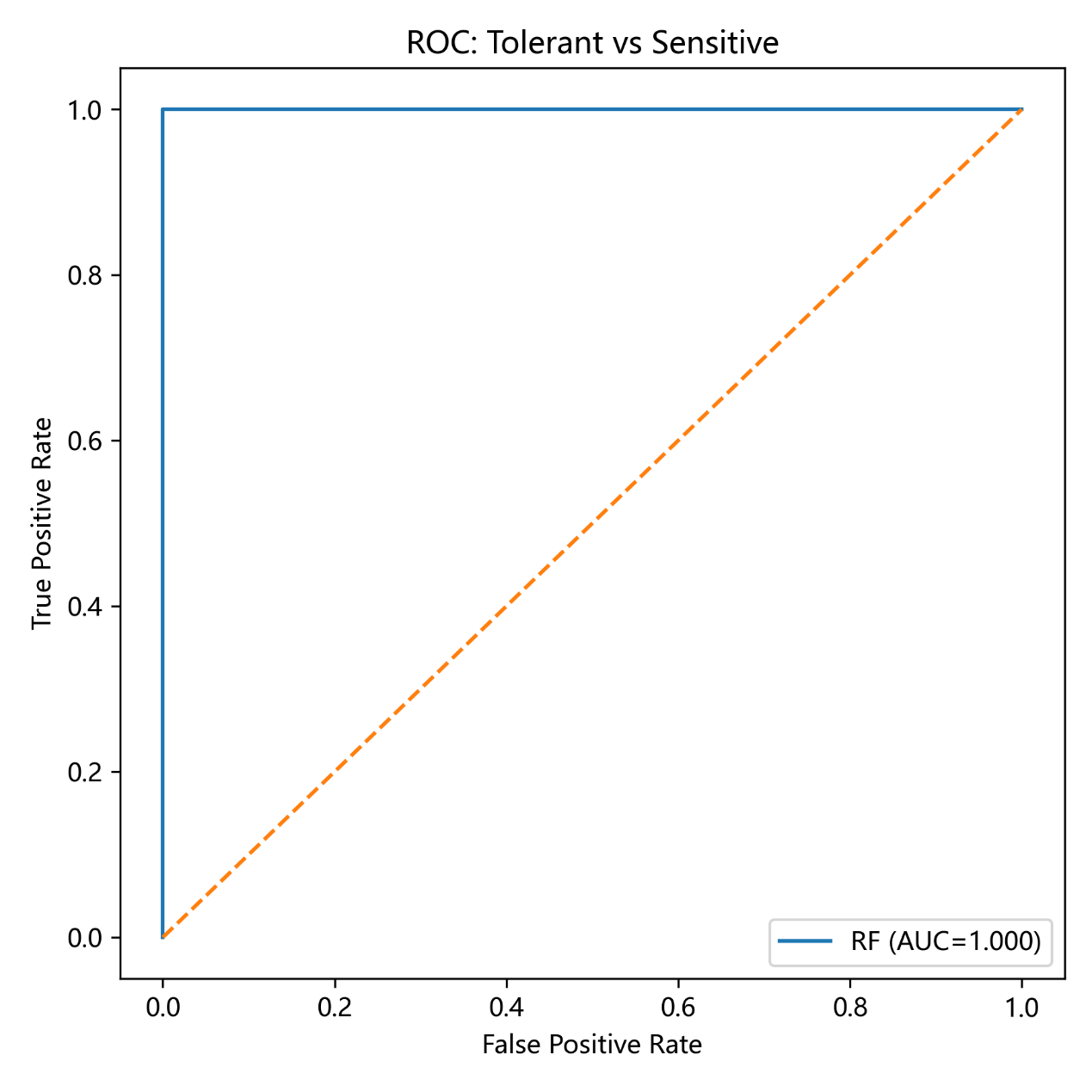


**Fig. S6** | ROC curves of a random forest model distinguishing between salt-tolerant and sensitive materials

Using the upper and lower quantiles of the STS (Sodium Salt Tolerance Scale) as salt-tolerant and sensitive categories, a random forest classification model was constructed based on the R-matrix phenotypic features. The ROC curves and their AUC values ​​were used to evaluate the model's discriminative ability. The results show that multiple phenotypic features can effectively predict the salt tolerance level of materials.


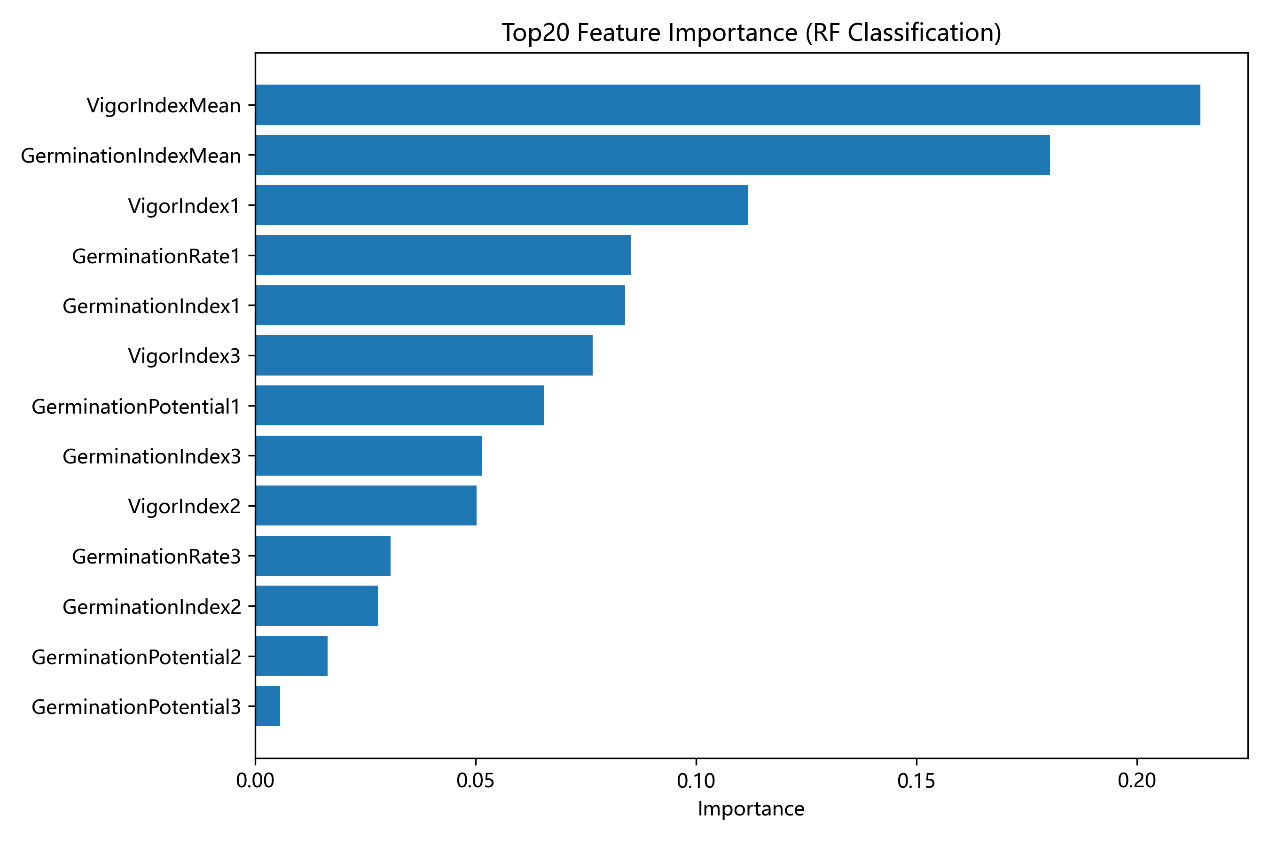


**Fig. S7** | Trait Importance Ranking (Gini Index) from Random Forest Model

Trait features are ranked based on the Gini importance output by the random forest model, showcasing key traits that contribute significantly to salt tolerance classification. This result is used to identify phenotypic indicators that play a dominant role in overall salt tolerance discrimination.


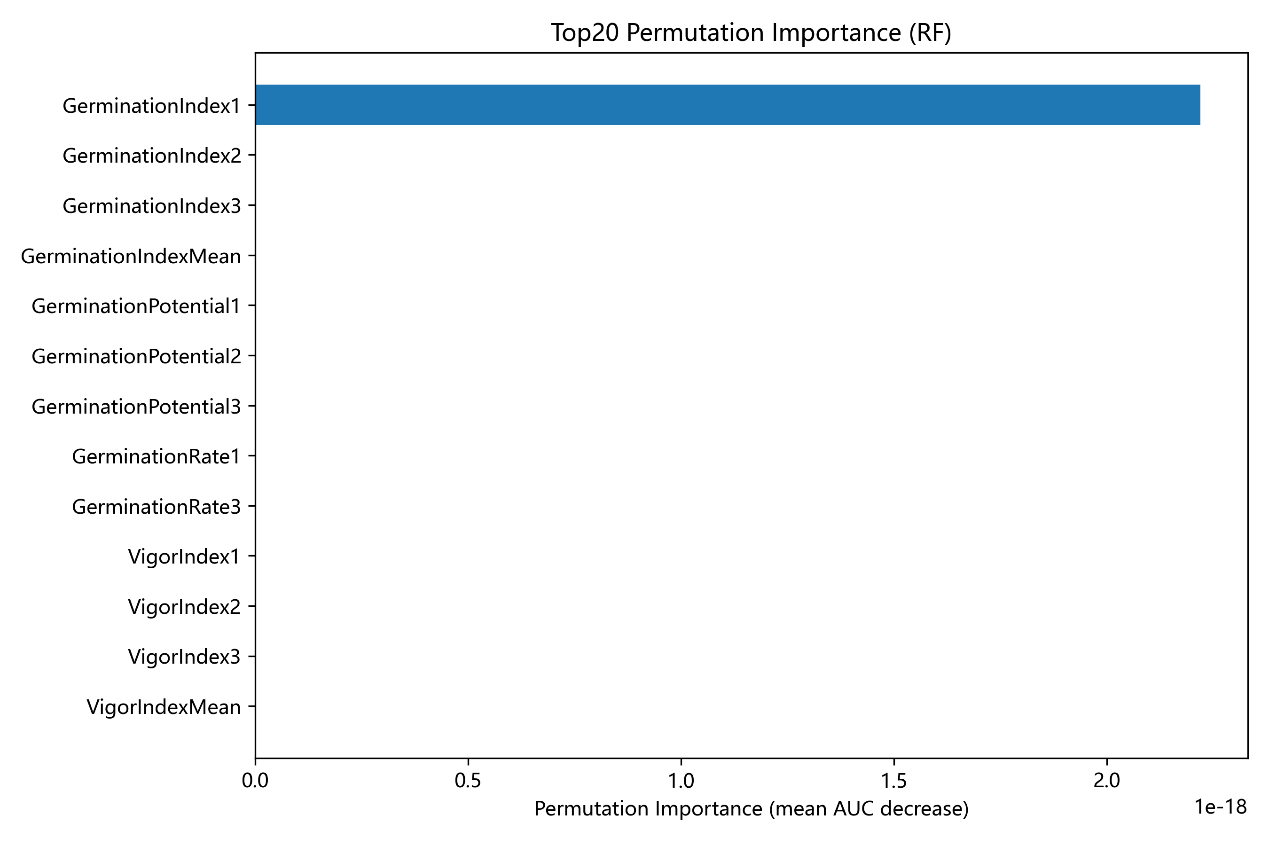


**Fig. S8** | Trait Permutation Importance Analysis Verifies the Robustness of Key Indicators

The permutation importance method was used to assess the impact of each trait on classification performance. The results were corroborated with Gini importance results to verify the consistency and robustness of key trait contributions under different assessment methods.


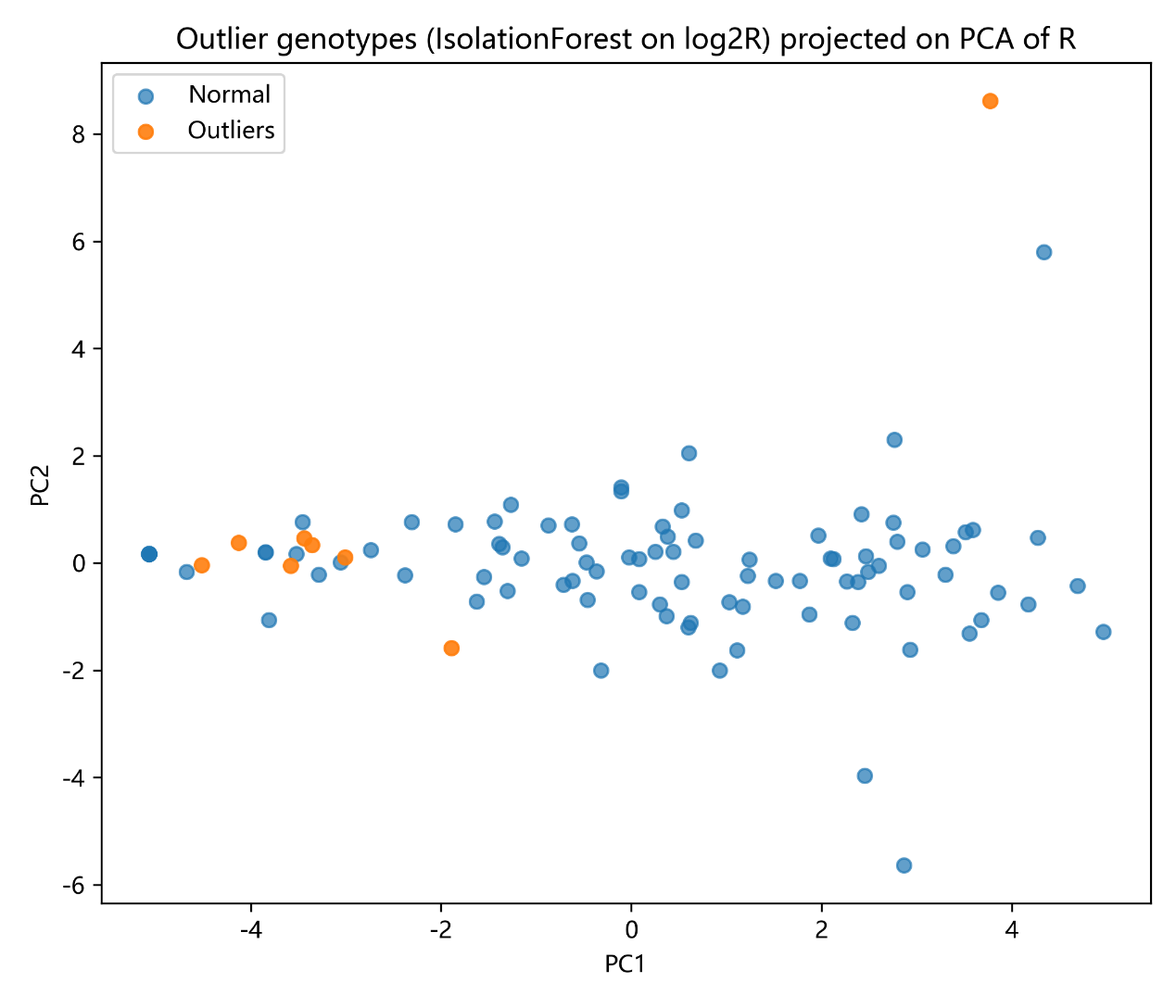


**Fig. S9** | Anomaly Material Identification and Analysis Based on PCA Space

Materials with anomalous response patterns were identified in PCA space using Isolation Forest based on log2R features. Anomalies represent materials that significantly deviate from the population trend under salt stress. This analysis provides candidate materials for identifying extremely salt-tolerant or highly sensitive germplasm.
