# Supplementary material for: Multilevel analysis of salt stress responses in sorghum during seed germination
Source: Front Plant Sci. 2026 Mar 19;17:1802398. doi: 10.3389/fpls.2026.1802398 (PMC13044168; doi:10.3389/fpls.2026.1802398)
Supplement: Supplementary file 2 [file Table2.doc]

**Table S1.** List of primers used in qRT-PCR.

| **S. No.** | **Gene** | **Forward primer (5’…3’)** | **Reverse primer (5’…3’)** |
| --- | --- | --- | --- |
| 1 | *SbNHXLP* | GGTGAACGAGTCCATCACCG | GAAACCCGGCATTGAAGATTATCGG |
| 2 | *SbMYBHv33* | GCTCTACAACCACATCAT | TAGTTGATCCACCGAAGT |
| 3 | *SbHKT1;4* | ATCGCCATCTGCATCACC | GCCTCCCAAAGAACATCACA |
| 4 | *SbTEF1* | GACCAGTCGGAGGACGAGAT | TCGCTGGATGGGTGCTTAC |
| 5 | *SbWRKY50* | CGATGACCTTCACCACTTCG | CTGAACCCGATCCTTCCACT |
| 6 | *SbActin* | TTCCAGCAGATGTGGATCTCCAAG | ATGTTTCTTCATGTAGAACATCGAT |
